# A written self-help intervention for depressed adults comparing behavioural activation combined with physical activity promotion with a self-help intervention based upon behavioural activation alone: study protocol for a parallel group pilot randomised controlled trial (BAcPAc)

**DOI:** 10.1186/1745-6215-15-196

**Published:** 2014-05-29

**Authors:** Paul Farrand, Claire Pentecost, Colin Greaves, Rod S Taylor, Fiona Warren, Colin Green, Melvyn Hillsdon, Phil Evans, Jo Welsman, Adrian H Taylor

**Affiliations:** 1 Mood Disorders Centre, School of Psychology, Washington Singer Laboratories, University of Exeter, Perry Road, Exeter EX4 4QG, UK; 2 University of Exeter Medical School, Heavitree Road, Exeter EX1 2LU, UK; 3 Sport & Health Sciences, University of Exeter, Heavitree Road, Exeter EX1 2LU, UK; 4 Lived Experience Group, Clinical Education Development and Research and Mood Disorders Centre, School of Psychology, Washington Singer Laboratories, University of Exeter, Perry Road, Exeter EX4 4QG, UK; 5 Plymouth University Peninsula Schools of Medicine & Dentistry, John Bull Building, Research Way, Plymouth, Devon PL6 8BU, UK

**Keywords:** Behavioural activation, Cognitive behavioural therapy, Depression, Paraprofessional, Physical activity promotion, Physical health, Pilot randomised controlled trial, Self-help

## Abstract

**Background:**

Challenges remain to find ways to support patients with depression who have low levels of physical activity (PA) to overcome perceived barriers and enhance the perceived value of PA for preventing future relapse. There is an evidence-base for behavioural activation (BA) for depression, which focuses on supporting patients to restore activities that have been avoided, but practitioners have no specific training in promoting PA. We aimed to design and evaluate an integrated BA and PA (BAcPAc) practitioner-led, written, self-help intervention to enhance both physical and mental health.

**Methods/design:**

This study is informed by the Medical Research Council Complex Intervention Framework and describes a protocol for a pilot phase II randomised controlled trial (RCT) to test the feasibility and acceptability of the trial methods to inform a definitive phase III RCT. Following development of the augmented written self-help intervention (BAcPAc) incorporating behavioural activation with physical activity promotion, depressed adults are randomised to receive up to 12 sessions over a maximum of 4 months of either BAcPAc or behavioural activation alone within a written self-help format, which represents treatment as usual. The study is located within two ‘Improving Access to Psychological Therapies’ services in South West England, with both written self-help interventions supported by mental health paraprofessionals. Measures assessed at 4, 9, and 12 month follow-up include the following: CIS-R, PHQ-9, accelerometer recorded (4 months only) and self-reported PA, body mass index, blood pressure, Insomnia Severity Index, quality of life, and health and social care service use. Process evaluation will include analysis of recorded support sessions and patient and practitioner interviews. At the time of writing the study has recruited 60 patients.

**Discussion:**

The feasibility outcomes will inform a definitive RCT to assess the clinical and cost-effectiveness of the augmented BAcPAc written self-help intervention to reduce depression and depressive relapse, and bring about improvements across a range of physical health outcomes.

**Trial registration:**

Current Controlled Trials ISRCTN74390532, 26.03.2013.

## Background

By 2020, depression is expected to become the second largest burden of disease globally [1], with up to one third of all depressed patients having episodes that last longer than 2 years [2]. Whilst evidence-based psychological therapies for depression exist [3], unfortunately, some three quarters of those successfully treated for depression will relapse, having at least one further depressive episode [2]. Depression and anxiety are estimated to cost the UK economy £17 billion in lost output and direct health care costs annually, with a £9 billion impact on benefit payments and lost tax receipts [4]. However, the potential costs of depression may be far higher since depression is associated with sustained physical inactivity [5,6] and consequently an increased risk of physical health co-morbidities such as obesity [7,8], diabetes [9], and stroke [10].

Links between depression and physical health [11], and the additional benefits of physical activity (PA) for depression beyond those provided by medication or therapy alone are well established [12]. However, health care services continue to reflect a ‘dualist’ philosophy [13], targeting mental and physical health separately. In a systematic review [14] examining the effectiveness of psychological therapies for treating depression, none of 204 trials reported a measure of PA, at baseline or follow-up. Therefore, little is known about the impact that changes in PA have upon improved mood. Whilst a small number of studies have sought to compare psychological interventions with exercise interventions [15,16], there have been few attempts to integrate PA into evidence-based psychological therapies for depression.

Behavioural activation (BA) potentially represents a key psychological intervention that is capable of being adapted to additionally target physical health outcomes alongside depression. Adopting a systematic and graded approach, BA targets the inertia and behavioural avoidance that often accompany depression. This helps to overcome sources of negative reinforcement that have maintained avoidance whilst exposing the patient once again to sources of positive reinforcement existent within the environment [17]. Whereas different BA models exist, they share a common theoretical underpinning based upon a behavioural model of depression [18].

Results from several systematic reviews [19] have established BA as an evidence-based treatment for depression [3]. Equal effectiveness for the treatment of depression when compared with other psychological interventions, including the package of interventions delivered within cognitive behavioural therapy (CBT) have been reported [19,20]. There have also been proposals to deliver BA for mild-to-moderate depression within a written self-help format [17,19]. This BA format features prominently as a key low-intensity CBT intervention within the Improving Access to Psychological Therapies (IAPT) programme, implemented across England [21,22].

Whilst aiming to increase behaviour, the focus of BA is, however, not conventional upon systematically increasing PA in a manner that could improve physical health outcomes by adopting techniques associated with PA promotion [23]. However, a focus upon improving physical health outcomes within BA may be achieved with only a slight shift in emphasis. For example, following an initial focus upon improving mood, at an opportune moment, the emphasis could be widened to also target physical health outcomes by selectively reinforcing activities that require greater energy expenditure. This shift in emphasis could potentially be facilitated by the augmentation of BA with a model of health behaviour change, such as self-determination theory [24], to help develop behavioural self-regulatory skills.

PA, in the form of exercise, is an evidence-based intervention for depression in its own right [25] and has additional physical health benefits that medication and psychotherapy treatments do not provide when used independently. However, evidence comes from trials involving participants who may be more motivated to do structured exercise sessions, with intensive support. With technological advances, such as accelerometers to capture PA, there is increasing interest in focusing interventions on breaking up sedentary time and increasing overall daily PA to enhance both physical and mental health [26,27]. Such interventions may also have a greater appeal to patients with depression when compared with those involving recommendations for types of structured exercises [3]. Augmenting BA with approaches to facilitate PA could therefore potentially broaden the effectiveness of the intervention when compared to BA alone, and represents an opportunity to address both mental and physical health outcomes. Both BA and PA promotion also seem highly compatible in approach, employing techniques such as self-monitoring, goal setting, and problem solving. All of these techniques have been independently shown to be effective behavioural change strategies [28].

Combining BA with PA promotion may also help to overcome limitations associated with the use of each intervention when used independently. In particular, the combined intervention may help to reduce the rate of depressive relapse that can occur following treatment. For example, following BA alone, up to two thirds of patients recovering from one episode of depression will relapse and have at least one further episode within 12 months [29]. Previous research examining the benefits of PA for depression raise the possibility that augmenting BA with PA promotion may help to reduce depressive relapse and maintain the therapeutic gains arising from the initial use of BA. For example, following successful treatment for depression, relapse rate 6 months post-treatment was 38% following initial treatment with medication alone, reducing to 31% with medication combined with PA and reduced further to 8% with PA alone [30].

### Study aims and objectives

Informed by the new Medical Research Council (MRC) framework for the development of complex interventions [31], the protocol for a phase II pilot randomised controlled trial (RCT) is reported. This study will examine a number of methodological uncertainties to inform the conduct of a phase III RCT to assess the clinical and cost-effectiveness of a combined BA and PA promotion intervention (BAcPAc) to reduce depressive relapse and improve physical health outcomes for adults with depression compared to BA alone, which represents treatment as usual. No prospectively-identified criteria for progression to a main trial have been set.

This pilot RCT does not involve any hypothesis testing and therefore there is no primary outcome. We are interested to test recruitment and retention rates as well as gain qualitative feedback on feasibility and acceptability of the methodology. The specific feasibility outcomes with the method of evaluation for this pilot RCT are shown in Table 1.

**Table 1:** Feasibility outcomes and evaluation

**Feasibility outcomes**	**Evaluation**
1. Feasibility of participant recruitment	Numbers assessed for eligibility, numbers eligible, reasons for ineligibility, reasons for non-participation, and numbers randomised
2. Appropriateness of data collection processes and outcome measures	Number of missing items and follow-up rates
3. Participant understanding of study information	Qualitative process interviews with participants following participation
4. Estimation of sample size to inform a phase III RCT	Differences at follow-up between study arms in the number of participants that reach an ICD-10 diagnosis of depression using the CIS-R [32]
5. Level of study attrition	Study and treatment drop-out rates
6. Estimate of the resources and costs needed to deliver the intervention	Variability in the number, length, and frequency of support sessions provided by psychological wellbeing practitioners
7. Acceptability towards the cognitive behavioural therapy self-help intervention received, and engagement with the BAcPAc intervention	Qualitative process interviews undertaken with participants following participation

## Methods

This protocol is informed by SPIRIT [33] guidance for the reporting of protocols of clinical trials (Additional file 1).

### Study design

**Figure 1:**
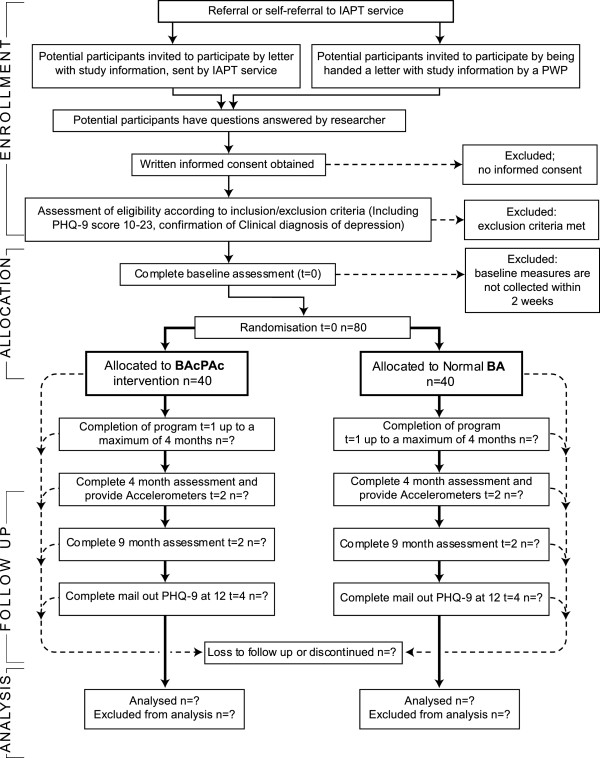
Consort diagram.

This study employs a single (researcher) blind, parallel-randomised controlled trial design with nested mixed methods process evaluation. Subjects are randomised individually in a 1:1 ratio to either a written self-help intervention based upon BA combined with PA promotion (BAcPAc) or a written self-help intervention based upon BA alone, representing treatment as usual (TAU) (Figure 1) [34].

Participants are being recruited through two IAPT services [21] located in one rural (East and Mid Devon) and one city based location (Plymouth). Both services are located in the county of Devon, South West of England.

### Participant inclusion criteria

Eligible participants are aged 18 and over, with a current episode of depression confirmed using the structured diagnostic Clinical Interview Schedule (CIS-R) [32] and score between 10 and 23 on the Participant Health Questionnaire (PHQ-9) [35] reflecting moderate to moderately-severe depression. Additionally, eligible participants are required to confirm that they can walk continuously and unaided for 5 minutes. Reflecting standard clinical practice, participants are eligible to participate in the study regardless of whether they are currently receiving antidepressant medication, as long as the dose has been stable for at least 28 days prior to recruitment into the study.

### Participant exclusion criteria

Potential participants are excluded from the study if they are currently receiving formal psychotherapy for their depression, been diagnosed with a severe and enduring mental health problem, are currently doing at least 30 continuous minutes of moderate intensity physical activity on 5 or more days of the week, score 2/3 on the PHQ-9 [35] suicide risk question, have been prescribed an antidepressant in the month prior to recruitment, have current substance or alcohol addiction, are unable to use written self-help materials in English, or are currently involved in another research study that would make participation difficult.

### Recruitment settings and procedure

Different recruitment strategies have been adopted for the two IAPT services participating in the study to allow comparison of different participant recruitment strategies. In Plymouth, potential participants meeting the inclusion criteria are invited to participate in the study at the end of the initial assessment session by being handed a study invitation pack by their psychological wellbeing practitioner (PWP) undertaking the assessment. In East and Mid Devon, the study invitation pack is posted to all potential participants who have self-referred or have been referred by a health professional into the IAPT service. In both cases, the invitation packs include a study invitation letter, study information sheet, and consent form.

### Screening, baseline, and informed consent

Potential participants willing to consider participation are asked to contact the researcher directly by phone or mailing the consent form (Additional file 2) with their contact details. Any further questions about the trial are answered on the phone and, if participants wish to participate, they are asked to provide verbal consent for a telephone screening informed by the inclusion criteria to assess eligibility for study inclusion. Following screening, and after gaining written consent, eligible participants are invited to attend a full baseline appointment with the researcher using the CIS-R [32] to confirm a diagnosis of major depression with participant randomisation undertaken following baseline assessment.

### Confidentiality

All personal data held electronically is stored on a password protected computer at the University of Exeter. Personal data held on paper files are locked in a filing cabinet at the University of Exeter, with the researcher being the only person with access to the key. All the information will be safely stored in line with the principles of the Data Protection Act. PWPs operating within the respective IAPT Services will follow guidance for Good Clinical Practice. Consistent with good clinical practice and detailed in the consent form, significant suicidal risk identified will be discussed with the participant and the GP will be informed. The consent form will also let the person know that, if we are very concerned about their safety or someone else’s safety, we would need to break confidentiality.

### Randomisation

A web-based minimisation service supported by the Peninsula Clinical Trials Unit ensures concealment allocation of participants to a PWP who has either been trained to deliver BA as usual or BAcPAc. To preserve researcher blinding regarding the study arm participants are randomised into, the Peninsula Clinical Trials Unit sends an e-mail containing randomisation details for the participant to the IAPT service administrator and a PWP supporting the intervention within that service. At the same time, the participant’s ID number with no other personal information is also e-mailed to the researcher.

Minimisation is based on the following prognostic factors: participant age (10–30 years or >31years), sex (male or female), PHQ-9 [35] score (moderate/moderately-severe 10–18; moderately-severe/severe 19–23), current use of anti-depressant medication (yes or no), and recruitment site (Plymouth or East and Mid Devon). To maintain concealment, the minimisation algorithm retains a stochastic element, providing a degree of unpredictability during the allocation process. The first participant was randomised on the 23^rd^ of April 2013.

### Sample size

A total of 80 participants (40 participants per arm) will be recruited. This sample size has been informed by the recommended sample size of ~30 participants per arm for estimation of outcome variance [36] and takes into account a 20% attrition rate over the duration of the study.

### Treatments

For both the BAcPAc (intervention) and BA alone (TAU) study arms, the BA protocol was developed by Richards [17] and represents the protocol implemented as part of the IAPT programme [21,22]. In the BA alone study arm, the BA protocol [17] has been directly transferred into a written self-help format specifically developed and adopted for use within the study (available on request from the authors). The patient works through the written self-help intervention with a maximum of 12 support sessions provided by a PWP [37]. In the BAcPAc study arm, the BA protocol [17] formed the foundation of an extensive development process based upon focus groups and informed by intervention mapping techniques [17] to deconstruct the BA protocol, and then to reconstruct it within a written self-help intervention [37] incorporating additional PA promotion techniques (available on request from the authors).

Interventions are delivered by four PWPs in both recruitment sites, with two randomised to deliver the BAcPAc study arm and two BA alone in both sites. The PWP role was developed as part of the IAPT programme to support low-intensity CBT interventions within a stepped care model of service delivery for patients presenting with depression and anxiety disorders (excluding PTSD and social anxiety) of mild-to-moderate severity [37]. All PWPs involved in the study have successfully completed an accredited PWP training programme based upon the curriculum [38] developed for the IAPT programme.

Furthermore, study PWPs also received an additional one-day training session delivered by members of the research team. The training introduced the PWPs to the study and provided them with the opportunity to address any questions. Additionally, procedures for working within the trial, including recording sessions and maintaining records on patient contacts, were reviewed. The training additionally overviewed the self-help intervention and theoretical rationale, appropriate to the study arm they were randomised to support. For PWPs in both study arms training used role-play to enhance skill development in supporting the appropriate self-help intervention. For PWPs supporting the BAcPAc intervention this also included skills enhancement in PA promotion techniques (Figure 1), included within the self-help intervention. Training sessions also provided the opportunity to ask any questions concerning the intervention protocols. PWPs supporting BAcPAc (Additional file 3) and BA alone (Additional file 4) are given a training manual to supplement the training received. During the course of the study, PWP supervision was conducted in accordance with IAPT supervision guidance [39]. Supervisors in the BAcPAc arm have also been provided with the BAcPAc intervention and encouraged to contact a member of the research team to answer any questions about the intervention or to raise questions emerging from the supervision sessions.

As with any pragmatic study, interventions in both study arms are delivered in accordance with the IAPT service delivery protocol developed to inform the delivery of low-intensity CBT interventions, including CBT self-help [37]. Consistent with this protocol, the number and length of support sessions in both study arms is not specified in advance, but determined collaboratively between the PWP and patient on the basis of any change in the patient’s depressive symptoms. Alternatively, participants are free to stop treatment or withdraw from the study at any time. In the case of a participant disclosing immediate suicidal risk to a PWP or researcher, the risk protocol will be followed and the participant will be withdrawn from the study.

Typically, participants receive one assessment session of up to 35 minutes followed by up to 12 support sessions of 25–35 minutes [37], with an average of 5.5 used within the IAPT programme [22]. The assessment session largely comprises a client-centred assessment to understand the difficulties experienced by the participant, administration of questionnaires to measure severity of depression, anxiety, and impact of mental health difficulty on work and social adjustment, alongside the provision of information concerning the wider impact of depression. Additionally, during the assessment session, participants are introduced to the written CBT self-help intervention related to the study arm they have been randomised into. Participants in the BAcPAc arm are given the BAcPAc written self-help intervention [40], whilst those in the BA alone arm are given the BA written self-help intervention [41]. Participants in both study arms are also given written gender-specific case studies that introduce the BAcPAc or BA alone intervention.

Subsequent support sessions are based around identifying the participant’s progress on the use of the specific written CBT self-help intervention developed for their study arm and provide guidance and encouragement to support the participants’ continued usage of the written self-help intervention. In the event the participant struggles with any activity detailed in the written CBT self-help intervention, the PWP reviews the intervention with the participant to enable him/her to problem-solve the difficulty and provide motivation to enable them to move forward with the intervention before the next support session. Dependent upon participants’ preference, support is provided face-to-face, over the phone, or through a combination of both methods. Furthermore, if requested, face-to-face sessions take place in a variety of locations including the participating IAPT service or GP surgery attached to the IAPT service.

#### Control arm

Representing treatment as usual, the intervention is delivered in accordance with the BA protocol [17], the IAPT service delivery protocol [37], accredited training received during their PWP training programme [38], and the one day training delivered by the research team to introduce the intervention and case studies in the written self-help format developed for the study.

#### Intervention arm

**Figure 2:**
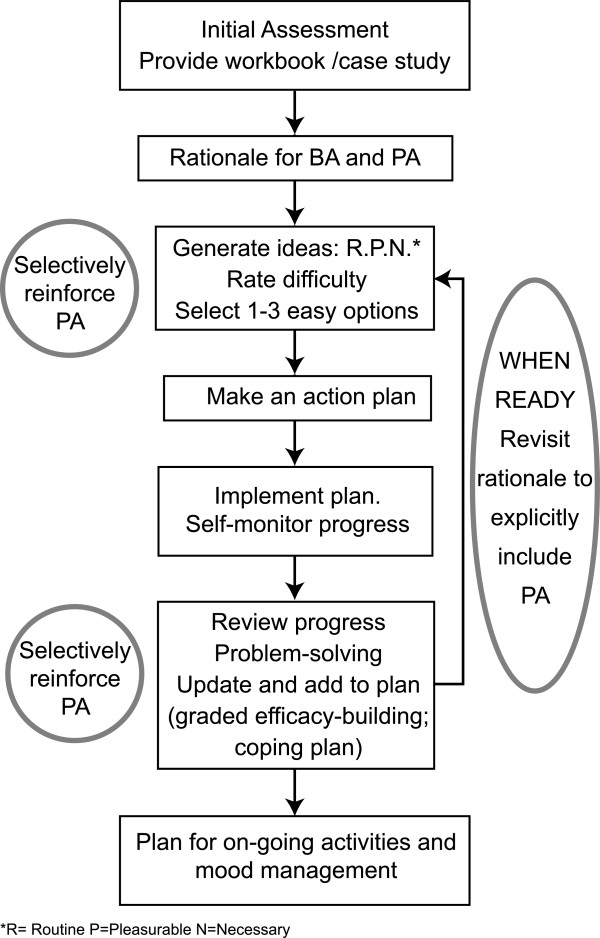
Intervention processes.

The written BAcPAc self-help intervention is identical to the self-help intervention used in the control arm with the exception of the following adaptations made to include targeted PA promotion elements (Figure 2).

i) The rationale for the intervention is altered to emphasise and reinforce the added value of activities that include a PA component.

ii) Principles derived from self-determination theory [24,26] are used to inform the development of the BAcPAc self-help intervention. In particular, this was used to adapt the text and content of the support provided by the PWP to help motivate and engage participants to maintain and develop plans to initiate more activities that involve sustainable PA into their normal weekly planning.

iii) If the PWP identifies participant mood starting to improve during treatment he/she encourages the participant to further increase PA using principles informed by self-determination theory [24] alongside techniques derived from motivational interviewing [42]. This helps maintain participant motivation to continue with the PA identified as part of the standard BA aspect of the intervention.

iv) The diary keeping elements of the BAcPAc self-help intervention were adapted to specifically prompt the participant to continue to monitor and plan their PA using the worksheets included within the intervention.

v) On occasions when the participant has planned PA involving walking within their diary, the PWP encourages the participant to use a pedometer [43] and record the number of steps taken per day on the appropriate worksheets included within the BAcPAc self-help intervention.

vi) Finally, unlike participants in the control arm, those randomised to the BAcPAc arm receive a 10–15 minute follow-up phone call by the PWP one month after the end of treatment at a time agreed between the PWP and participant during the last support session. The aim of this appointment is to review progress with the BAcPAc self-help intervention and support the participant to continue with PA by encouraging them to continue to self-monitor PA using diaries and/or pedometers, problem-solve any difficulties, and resume the BAcPAc self-help intervention and case studies if they identify any worsening of mood.

### Blinding

Participants and research staff conducting assessments are kept blind to study arm allocation as far as possible. Throughout duration of the trial, participants are reminded not to disclose the study arm they have been randomised to during contact with the researcher. A member of the research team collects research data, with blinding concerning participant study arm allocation maintained as far as possible. A researcher (CP) checks all data for accuracy before conversion to electronic format using TeleForm software (http://www.cardiff-teleform.com). Cardiff TeleForm is programmed to perform a range checks for numerical data and to inspect every data entry point is present. All data collected from participants will be assigned a unique ID number and kept separate to any patient identifiable data. All data is kept in lockable cabinets at the University of Exeter, and is not shared with anyone outside the research team. Statistical analyses are undertaken by a member of the research team (FW) blinded to study arm allocation. Instances that arise regarding accidental disclosure of allocation are recorded by the unblinded research team member.

### Outcome measurement

#### Feasibility outcomes

The feasibility outcomes are related to the aims associated with a pilot RCT [44]. Descriptive comparisons across the primary outcome measures of interest will be made between the two recruitment sites adopting different recruitment techniques. The feasibility outcomes are outlined in Table 1.

#### Assessments

To inform inclusion within a definitive phase III RCT, data on the following assessments will be collected.

i. Diagnosis of depression: ICD-10 diagnosis as a binary (yes or no) outcome by the CIS-R [32].

ii. Symptoms of depression and index of depressive relapse: PHQ-9 [35] based upon an adapted protocol [45].

iii. PA sedentary behaviour and total energy expenditure: assessed using an accelerometer (GENEActiv) worn on the wrist for one week and a 7-day PA recall survey [46].

iv. The Insomnia Severity Index (ISI) [47].

v. Health-related quality of life: Short Form-36 (SF-36 [48]) and EuroQol (EQ-5DL [49]).

vi. Health and social care services use and cost: simplified version of the Adult Service Use Schedule (ADSUS [50]).

vii. Blood pressure: the average of the last two from three measurements taken seated from the left arm following 10 minutes of rest measured using an Omron M7 Monitor.

viii. Body mass index: weight measured using Omron HN286 Digital Scales and height measured using a tape measure.

In addition, general demographic information, including gender, age, ethnicity, marital status, smoking status, postcode, number of dependants, and age of leaving education, is collected from all participants at baseline.

### Data collection

The researcher collects general demographic information during telephone screening. All other data informing the feasibility outcomes is extracted from routine service records by the PWP delivering the intervention at the end of the support session, by the researcher during 4-, 9-, and 12-month follow-up, or from process interviews. All participants will be followed-up despite any deviation from planned treatment. Time points representing collection of the assessments are highlighted in Table 2.

**Table 2:** Assessments administered at screening, baseline, and follow-up post-baseline

	**Screening**	**Baseline**	**4 months post baseline**	**9 months post baseline**	**12 months post baseline**
PHQ-9	X		X	X	X
CIS-R	X		X	X	
Physical activity					
7 day PA recall		X	X		
Accelerometer			X	X	
Body mass index		X	X	X	
Blood pressure		X	X	X	
Health and social care service use (ADSUS)		X	X	X	
Sleep-insomnia severity index		X	X	X	
Quality of life					
SF-36		X	X	X	
EQ-5D		X	X	X	

Assessments taken at baseline are collected face-to-face or over the telephone depending upon patient preference, with height, weight, and blood pressure collected face-to-face. All data collected at 4-month follow-up is collected face-to-face, with 9 and 12 month follow-up data collected face-to-face or over the phone, depending on patient preference. Adverse events are reported by PWPs to the researcher during routine communication and spontaneously. The researcher inquires of any adverse events at follow-up. Any events are recorded and escalated to the PI, and if necessary, to the sponsor.

### Process evaluation

The aims of the process evaluation are to assess or identify: i) the acceptability of the BAcPAc intervention for patients and PWPs, ii) the fidelity of delivery of the BAcPAc intervention (how well does delivery match our intentions), iii) additional training needs for PWPs delivering the interventions, iv) any future adaptations needed in the BAcPAc intervention. Data gathered as part of the process evaluation will be used to further adapt the BAcPAc written self-help intervention and training in preparation for the phase III RCT. Qualitative interviews are conducted with participants randomised to the BAcPAc study arm and with PWPs supporting the interventions.

#### Qualitative interviews

After the final support session, all participants receiving the BAcPAc intervention are invited to participate in a semi-structured interview. This examines intervention acceptability and identifies adaptations that participants feel may improve the assessment or support provided for the BAcPAc intervention. Interviews follow a predetermined interview schedule, with the topic guide (Additional file 5) partially informed by a previous qualitative study investigating acceptability of CBT self-help for the treatment of depression in patients with chronic physical illness [51]. This was developed in consultation with the intervention development team and a patient and public involvement group specifically established to support the study. Interviews are digitally recorded and transcribed verbatim. Data is analysed using framework analysis [52] alongside constant comparison techniques [53] to generate concepts and themes. To ensure rigour, the researcher reads transcripts and develops the initial categorisation, with second coding undertaken by another member of the research team. Further categorisations are discussed amongst the team and amendments to categories and definitions made as required [54]. Qualitative data is analysed using ‘surface level’ thematic analysis only. This involves coding the data to extract concepts and grouping the concepts together into themes.

#### Intervention fidelity checking

All support sessions (face-to-face or telephone) in both study arms are digitally recorded. A 20% sample is then assessed to review intervention fidelity for each PWP using an intervention fidelity measure. Recordings are listened to by a member of the research team, who applies the intervention fidelity measure to rate competence in each element of the intervention. Based on previously used intervention fidelity measures [26,55] and informed by a prior intervention mapping procedure [56], a version specifically developed for the BAcPAc intervention (Additional file 6) was adopted. The measure incorporates the Dreyfus skill acquisition scale [57] to rate the competence of providers in delivering the targeted techniques and delivery processes. The scale produces a score of 0 to 6 for each targeted element of the intervention process and is anchored so that a score of 3 is considered acceptable, 0 is non-existent, and 6 is perfect performance. Intervention fidelity measure scores are summarised using simple descriptive statistics (means and standard deviations) and collated both in total and by therapist. Examples of good practice are flagged, transcribed, and extracted as audio clips or text transcripts to inform future training. Items scoring low for some or all PWPs supporting the intervention are used to identify areas where change is needed to the intervention materials or PWP training course.

### Statistical analysis

#### Quantitative

For both recruitment settings, participant flow will be summarised using the CONSORT flow diagram adapted for complex interventions [58], reporting recruitment and attrition rates (both treatment and study drop-outs) with 95% confidence intervals. The CONSORT flow diagram will also be adapted to reflect the number of recruitment letters sent, numbers consenting, number randomised, number undertaking intervention, and number of completed outcomes alongside means, standard deviations and frequencies regarding the number of support sessions, session length, and mean number of days between support sessions. All protocol deviations, along with number of missing items on questionnaires, will be reported. Mean and standard deviations for general demographic information will be reported at baseline with assessments conducted at baseline and 4-, 9-, and 12-month follow-up.

#### Economic analysis

Factors related to collecting self-reported data on participant NHS and related resource use will be examined. Informed by case report forms, methods for estimating the resource use and costs associated with delivery of the intervention will be developed and tested. Whilst it is not possible to estimate cost-effectiveness in this pilot study, estimates of intervention cost will inform exploratory modelling and estimates of the resources and costs needed to run a future trial. In a future phase III RCT, cost-effectiveness analysis is expected to involve both within-trial analysis and evidence synthesis with decision-analytic modelling, to assess the longer-term consequences of the intervention (e.g., changes in outcomes, events avoided, life-years, and quality-adjusted life-years gained). It is expected that this modelling will use/adapt existing economic models on outcomes for depression and PA promotion, and the subsequent cost-effectiveness of interventions. This study will be used to conduct preliminary investigations on how best to construct such analyses and develop an economic evaluation plan for the planned phase III RCT. A summary of the results will be available to participants who have requested this during consent, and a report provided to the participating IAPT services. The findings will be reported in a number of peer reviewed journals.

### Ethical approval

This protocol has been reviewed and approved (26/03/2013) by the NHS South West Regional Ethics Committee (REC reference number: REC/SW/0291).

### Changes to methods since recruitment began

Since trial recruitment began in April 8^th^ 2013, the following important changes have been made:

1) A small number of potential participants from Mid and East Devon had reported that they would experience difficulties taking part in the study if they could not be seen at a convenient location. Originally, PWPs were only seeing participants in one location. We therefore extended the number of locations where patients could be seen for treatment and, as the service routinely offer face-to-face or phone appointments to patients, we also changed the information sheet to emphasise that participants entering the study could be treated either face-to-face or over the phone. (Approved 15/08/2013)

2) Participants often have had their prescription changed by their GP at the same time as referral to the service, and so we were excluding many potential participants. This has been considered useful for previous trials to establish stability in mood prior collecting baseline measures, but is not a clinical requirement. Given a high rate of exclusion from this study, we are now recording if participants have changed or been prescribed an antidepressant in the last month, and are collecting information about current and past medication from all participants at baseline and follow-up. We have therefore removed the exclusion criteria “changed or newly prescribed antidepressant in the previous month”. (Approved 15/08/13)

## Discussion

This phase II pilot RCT has been designed to answer important methodological uncertainties, the answers to which can be used to inform the design and implementation of a planned phase III RCT. Undertaking pragmatic trials in natural settings are notoriously complex and require careful testing to determine the acceptability and effectiveness of the trial methods, including those associated with the intervention. The need for this phase II RCT is especially important given that it is proposed to locate the planned phase III RCT within IAPT services with the intervention delivered by a paraprofessional PWP workforce. To date, both the setting and workforce have been employed within few RCTs [59].

Furthermore, given the novel basis of the written BAcPAc self-help intervention, this study also includes a planned process evaluation and assessment of intervention fidelity to provide valuable information on how to refine the written BA self-help intervention to include PA promotion techniques. When best to emphasise these techniques within the support protocol following improvements in mood associated with an initial emphasis upon BA will also be addressed. There is currently no information on whether patients who receive BA spontaneously become more physically active and the data collected in this study will help to inform the extent that any adapted BAcPAc intervention would need to increase PA in order to have any impact on physical and mental health outcomes in a definitive trial.

Combining BA with PA within the BAcPAc written self-help intervention offers the potential to increase time to depressive relapse, provide patients an additional method to manage their depression, and lead to physiological health benefits resulting in reduced risk for physical health co-morbidities. Development and evaluation of such an intervention is timely given proposals to expand the competencies associated with the PWP role to include an increased emphasis upon physical health [60]. If effective, such an intervention also provides the potential to target mental and physical health outcomes within a single intervention and further increase access to evidence-based psychological therapies [21,22].

## Trial status

The recruitment phase of this study started in April, 2013 and has an intended completion date for recruitment of April, 2014.

### Access to data

The full cleaned dataset will be held by Dr Paul Farrand at the University of Exeter. All investigators will have full access to the data after a formal request describing their plans is approved by the steering group.

## Abbreviations

BA: Behavioural activation; BAcPAc: Behavioural activity and physical activity promotion; CBT: Cognitive behavioural therapy; CIS-R: Clinical interview schedule; IAPT: Improving access to psychological therapies; PA: Physical activity; PWP: Psychological wellbeing practitioner; RCT: Randomised controlled trial.

## Competing interests

The authors declare that there are no competing interests.

## Authors’ contributions

All authors contributed to the design of the study and the drafting of the manuscript, with no professional writers employed. All authors read and approved the final manuscript. PF: conception and design, intervention development, manuscript writing, and final approval of the manuscript. CP: intervention development, data collection and analysis, manuscript writing, and final approval of the manuscript. CGrvs: intervention development, intervention mapping, critical revision, and final approval of the manuscript: RST: design, data analysis, critical revision, and final approval of the manuscript. FW: data analysis and final approval of the manuscript. CGrn: methods for the cost-effectiveness component, final approval of the manuscript. MH: conception and design and final approval of the manuscript. PE: primary care expertise and final approval of the manuscript. JW: service user perspective, data collection, and final approval of the manuscript. AT conception and design, intervention development, manuscript writing, and final approval of the manuscript. All authors read and approved the final manuscript with Authorship Eligibility Guidelines determined by SPIRIT adopted.

## Supplementary Material

Additional file 1 SPIRIT checklist. Click here for file

Additional file 2 Study consent form. Click here for file

Additional file 3 PWP training manual BAcPAc. Click here for file

Additional file 4 PWP training manual BA. Click here for file

Additional file 5 Participant topic guide. Click here for file

Additional file 6 BAcPAc intervention fidelity measure. Click here for file
