# Supplementary material for: A written self-help intervention for depressed adults comparing behavioural activation combined with physical activity promotion with a self-help intervention based upon behavioural activation alone: study protocol for a parallel group pilot randomised controlled trial (BAcPAc)
Source: Trials. 2014 May 29;15:196. doi: 10.1186/1745-6215-15-196 (PMC4061537; doi:10.1186/1745-6215-15-196)
Supplement: Additional file 2 — Study consent form. [file 1745-6215-15-196-S2.pdf]

A study for people with depression and low mood

Consent Item	Please Tick Yes	Please Tick No
I confirm that I have read and understood the participant information sheet for the study and have had the opportunity to ask questions.		
I understand that my participation is voluntary and that I am free to withdraw at any time, without giving a reason, without my usual care or legal rights being affected.		
I understand that I may not be eligible to take part in the study.		
I understand that details of my participation will be stored anonymously on file and may be used in the final analysis of data.		
I agree to take part in the study.		
I agree for my General Practitioner and [Depression and Anxiety Service] to be informed if my condition deteriorates in a way that there are concerns I may be suicidal or at significant risk of harm to myself or others.		
If I am experiencing problems or feel that something is going wrong with the study I realise I should contact Dr Claire Pentecost (University of Exeter, Mood Disorders Centre, Room 307, Washington Singer, Perry Road, Exeter, EX4 4QG; Telephone: 01392 724653; Email: c.pentecost@exeter.ac.uk) or one of the research team members.		
I agree to allow digital recordings of the support sessions with the PWP to be made.		
I agree for my GP to be informed of my participation in this study.<br>You do not need to tick yes to participate in this study.		
I would like to receive information about the findings of this study. I am happy for my contact details to be kept until after the study so I can be sent this information.<br>You do not need to tick yes to participate in this study.		

Print Name: ____ Tel. number: ________________________________

Signed: ____ Date: ______________________________________

Best day to call: ____________________________________ Best time of day: ____________________________

GP Name: __________________________________ GP Address: __________________________________

Signed (University of Exeter) ________________________ Date: _____________________________________

Print Name: ____
