# Supplementary material for: A written self-help intervention for depressed adults comparing behavioural activation combined with physical activity promotion with a self-help intervention based upon behavioural activation alone: study protocol for a parallel group pilot randomised controlled trial (BAcPAc)
Source: Trials. 2014 May 29;15:196. doi: 10.1186/1745-6215-15-196 (PMC4061537; doi:10.1186/1745-6215-15-196)
Supplement: Additional file 3 — PWP training manual BAcPAc. [file 1745-6215-15-196-S3.pdf]

UNIVERSITY OF EXETER

BAcPAc

Actively beating depression

A study for people with depression or low mood

BAcPAc Training manual for Psychological Wellbeing Practitioners delivering the BAcPAc intervention

BAcPAc Psychological Wellbeing Practitioner Research Manual
Version 2
February 2013
Claire Pentecost, Adrian Taylor, Paul Farrand and Colin Greaves
University of Exeter

Contents

The BAcPAc Project: Background 5

Introduction to BAcPAc 6

Study Aim 6

Study Objectives 6

What is Physical Activity? 6

Physical and psychological benefits of Physical Activity 7

Physical health benefits of Physical Activity 7

..... 7

Evidence of benefits of Physical Activity for depression 7

..... 7

Challenges of increasing Physical Activity for people with depression 8

How might Physical Activity enhance mood? 8

Mood regulation 8

Rumination 8

Identity Change 8

Connectedness 8

Gaps in the Evidence 9

How much Physical Activity should I be encouraging for optimum mood change? 9

The three C's 9

Confidence 10

Control 10

Connection 10

The BAcPAc intervention – Increasing Physical Activity within Behavioural Activation 11

Study rationale 11

Physical Activity and the Behavioural Activation Model 11

The BAcPAc Intervention 13

What are we asking PWPs to do? 13

Looking more closely at setting goals for physical activity 15

Frequency, intensity, timing and type: The F.I.T.T. principle 15

Sustainability and relapse 16

Using the BAcPAc self- help booklet and case study 16

Worksheet C: This is where participants can explore types of activities that should be realistic and hopefully sustainable 19

Final session 24
Follow up sessions 24
General clinical procedures 24
Frequency and duration of appointments 24
Collecting routine outcome measures 25
Recording sessions 25
Pedometers 26
Appointments with BAcPAc supervisor 26
Organisation and management 26
Risk protocol 26
Participants randomisation and research being blinded to group allocation 27
Confidentiality and data protection 28
Finally 28

The BAcPAc Project: Background

- By 2020 depression is expected to represent the second highest burden of disease among all general health problems.
- Three quarters of those successfully treated for depression will go on to have at least one more depressive episode.
- Depression and anxiety are estimated to cost the UK economy £17bn in lost output and direct health care costs annually, with a £9bn impact on the Exchequer through benefit payments and lost tax receipts.
- The potential costs of depression may be far higher with the financial burden associated with sustained inactivity that is a key symptom associated with depression, alongside an increased risk of health related problems such as obesity, diabetes, and stroke.
- Despite links between mental and physical health, interventions tend to target mental and physical health separately, with little guidance on how best to promote PA within mental health services.

IAPT services

- Psychological Well-being Practitioners (PWPs) are a new workforce created as part of the IAPT (Improving Access to Psychological Therapies) programme implemented across England.
- PWPs undertake a range of evidence based low intensity psychological interventions including Behavioural Activation (BA), which seeks to reduce depressed mood by increasing daily functioning.

Behavioural Activation

- BA is established as an evidence based treatment for depression.
- Systematic reviews have also demonstrated equal effectiveness of BA for the treatment of depression to other psychological interventions, such as cognitive behavioural therapy (CBT).
- BA seeks to reduce depressed mood by increasing daily functioning.
- BA is a technique where participants focus on re-establishing daily routines, increase pleasurable activities and do the things that are necessary for daily living.

Depression and Physical Activity

- Physical Activity has shown to reduce the time between treatment and relapse compared to both CBT and medication, or a combination of the two
- NICE recommends Physical Activity for people with depression, in the form of 45-60 minutes of structured supervised exercise on 3 occasions per week. This is based on studies with an aim to show that it can be beneficial but disregards the fact that it may be difficult to undertake this amount for most patients with depression. The benefits of Physical Activity for people with depression have also been promoted in the National Physical Activity campaign ‘Let’s Get Moving’.

- There are high rates of comorbidity between patients with depression, chronic disease and physical health decrements.
- Some evidence has shown that Physical Activity might be an effective treatment for depression (Mead et al 2009).
- Studies on PA and depression have mostly involved intensive non-generalisable interventions for the UK. Further pragmatic research is needed on how best to integrate Physical Activity into existing low intensity interventions.
- There is a need for new interventions that are more acceptable for patients with low mood, and build on existing evidence-based therapies such as BA.

Introduction to BAcPAc

Study Aim

To enhance a routine, evidence-based behavioural activation (BA) treatment for people with depression by adding a focus on Physical Activity (PA) behaviour change techniques in a novel intervention, and conduct a pilot RCT to inform the design and delivery of a larger scale randomised controlled trial (RCT).

Study Objectives

Phase II Pilot RCT: (a) Undertake a pilot RCT of BA alone vs. BAcPAc to confirm feasibility and clarify uncertainties in the design and delivery of a larger scale RCT; (b) Assess the acceptability of the BAcPAc intervention amongst participants with depression.

What is Physical Activity?

Physical Activity is any body movement that works your muscles and uses more energy than you use when you are resting. Exercise on the other hand is purposeful Physical Activity for health benefits. Promoting any Physical Activity fits well with Behavioural Activation. The PWP can support any Physical Activity tailored to Participant needs but it is expected to be mainly walking and daily Physical Activity, rather than structured exercise.

People with depression, even amongst those who are physically active, the term Physical Activity is more acceptable than the term 'exercise'. Some people have negative associations with the term 'exercise' as they think of their last experience of it – often at school on a cold football or hockey pitch!

Physical and psychological benefits of Physical Activity

Physical health benefits of Physical Activity

- Physical Activity has many physical health benefits. Such benefits include: reducing blood pressure, reducing body fat and circulating blood fat, reducing risk of diabetes, coronary artery disease, stroke and a number of cancers; and helping to manage chronic pain, asthma and diabetes.
- The risks of engaging in Physical Activity are low for most of the population, but the risks of poor health resulting from inactivity are high.
- People who are sedentary have more risk factors for a number of conditions than those who are physically active.

The risks associated with taking part in Physical Activity at a level that promotes good health are low (Let's Get Moving commissioning guidance).

Evidence of benefits of Physical Activity for depression

- Physical activity has been shown to reduce time to depressive relapse (Babyak et al. 2000)
- Depression and a range of poor health behaviours often coexist (Gagnes et al. 2002; Simon et al. 2008)
- Depression is associated with a range of health related conditions
  - Cardiovascular risk (Frasure-Smith et al. 2005)
  - Diabetes (Peyrot & Rubin, 1999)
  - Stroke (Jonas & Mussolino, 2000)
- NICE (2009) Guidelines for treating depression: Translating evidence to practice
  - Physical Activity programmes for people with persistent sub threshold depressive symptoms or mild to moderate depression should be delivered in groups with support from a competent practitioner
  - Consist typically of three sessions per week of moderate duration (45min-1hr) over 10-14 weeks
- Cochrane review (Rimer, 2012)
  - 30 trials (1858 participants) compared exercise with no treatment or a control group and the pooled standardised mean showed a moderate clinical effect.

"Physical Activity is effective in the treatment of clinical depression and can be as successful as psychotherapy or medication, particularly in the longer term" (Department of Health 2004)

Challenges of increasing Physical Activity for people with depression

These are some of the difficulties of engaging in regular Physical Activity for people with depression:

- Low self-worth/physical self-worth
- Tiredness
- High likelihood of other physical health issues

With a small increase in mood arising during the use of BA, we may expect that it will start to be a little easier to engage with Physical Activity. This may enable a person with depression to become ready to make small steps to increasing Physical Activity.

How might Physical Activity enhance mood?

There are several theories as to the mechanism for the relationship between Physical Activity and psychological well-being amongst people with depression. These include:

- Mood regulation
- Rumination
- Identity change
- Connectedness

Mood regulation

Physical Activity has an acute effect of releasing endorphins, and this can help to regulate mood.

Rumination

People with depression have reported that engaging in Physical Activity, especially with others or involving skill or concentration, helps to reduce rumination of negative thoughts.

Identity Change

How we behave, and our thoughts have an influence on how we see ourselves. Physical Activity is seen by most people as a positive, healthy activity, and participating in more Physical Activity can improve people's perceptions of themselves. Physiological responses to increases Physical Activity such as increased energy and weight loss or improved muscle tone, can improve self-perceptions of attractiveness.

Connectedness

Getting out and about into the world is part of increasing Physical Activity, and this often involves seeing and interacting with other people. We may start to engage in physical activities with other people. Social interaction is good for our health, people with low levels of social interaction suffer from more chronic conditions than those who have good levels of social interaction. A positive connection with our physical environment is also important for general health and well-being.

Gaps in the Evidence

- The challenge is how to support people with depression, who may have low confidence and energy, to become more physically active.
- Despite links between mental and physical health, there is little guidance on how best to promote Physical Activity within mental health services.
- We do not know exactly how mood is enhanced, or the dose required to have an effect (not addressed in this study).

How much Physical Activity should I be encouraging for optimum mood change?

There is no definitive answer to this question, as everyone responds to Physical Activity differently, and currently there is not enough evidence that has demonstrated a particular form or dose of Physical Activity is optimum for people with depression. However, we do know that the government promotes a specific amount of physical activity for general physical and mental health benefits.

Unfortunately, many people think that they are already active enough, when in fact they are not meeting the national guidelines for Physical Activity. Our recommendation is that ANY increase in regular Physical Activity will be better than staying the same.

Our challenge is to try and increase Physical Activity in people with depression in order that it can be sustainable. Therefore we would like to focus on QUALITY of the experience rather than have participants focus on the QUANTITY. Once they can see the value of their increased Physical Activity, then is the time to think more about quantity.

The three C's

There is evidence to suggest that there are 3 elements of an activity that together have an impact on the quality of the experience i.e. enjoyment and satisfaction. The 3 C's are:

- **Confidence**: activities you feel you can achieve and help to gain confidence in the activity
- **Control**: activities you feel you have some control over achieving
- **Connection**: activities that involve others (through sharing the moment or talking about the experience), and with pleasant environments (e.g., parks, countryside and coast)

Confidence

Participants need to feel that they 'can do' the activity. Increasing feelings of being able to do it can be through their own experience of having done it before, or through seeing or hearing about other people (especially people similar to them) who can do it. Activities that appear too difficult to start with will not inspire confidence.

Control

Activities that involve outside factors that are not in the persons control are likely to be more difficult. Also feeling a sense of control over deciding '**W**hat' activity to do, '**W**hen' to do it, '**W**ho' to do it with and '**W**here' it will take place will positively impact on the experience.

Connection

Having support to do Physical Activity, either by exercising with others, or being positively verbally supported by others can help to see value in the experience. For many the connection with the environment is also important, whether that the garden, the park, in the countryside somewhere or by the beach.

Building a sense of confidence and control are already inherent in the Behavioural Activation treatment method. For example, PWPs will already be building confidence through helping patients with careful realistic goal setting, and being patient centred in approach will help patients feel they have a sense of control. For BAcPAc we are therefore also trying to ensure that 'connection' is considered when goal setting. However, not everyone will want or need to exercise with others or to have others involved in their physical activity – some may even be put off by negative experiences of other peoples involvement. It is worth exploring with each participant how their enjoyment and satisfaction could be maximised by considering the 3 C's.

It is important to reiterate that mental health benefits from participating in Physical Activity are not only likely to be associated with the amount of Physical Activity (the duration and how hard the individual is working) but also the type of activity (alone or with someone else, the environment the activity takes place, and the form of the activity). Some people may feel uplifted after going for a brisk walk on the local beach for 30 minutes, someone else might feel better after meeting a friend to go swimming.

The BAcPAc intervention – Increasing Physical Activity within Behavioural Activation

Study rationale

Physical Activity and the Behavioural Activation Model

As you know, Behavioural Activation is based on the knowledge that depression has an effect on:

1. Things we feel physically
2. Things we do or stop doing
3. Things we think

For people with depression, BA seeks to address the things that we do in order to influence physical symptoms such as tiredness, problems sleeping, appetite; and to reduce thinking guilty or worthless thoughts, and low confidence. With the added emphasis on 'Doing' *physical* activities we are focusing on a particular form of 'doing' that has an influence on a) physical feelings and b) thoughts, but also has been shown to reduce time to relapse for people with depression, and has important physiological benefits.

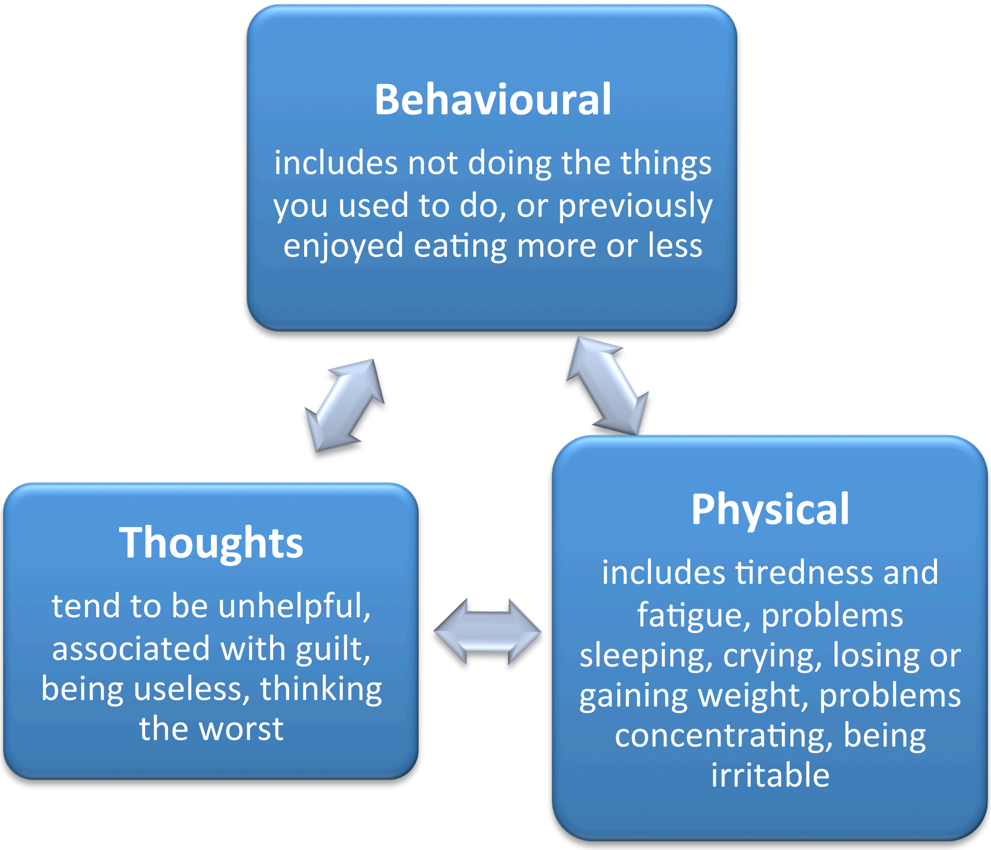

Depression affects our thoughts, our behaviour and our physical self and each of these can moderate the other.

We wish to add to this model by showing the additional benefits to adding and increasing the amount of PHYSICAL activities. The diagram below shows the cycle of low mood with an emphasis on the effects of reduced activities, which when reversed, will have benefits in areas such as reduced stress, increased energy, improved sleep, increased concentration, improved health (blood pressure, weight) and provides a distraction from rumination.

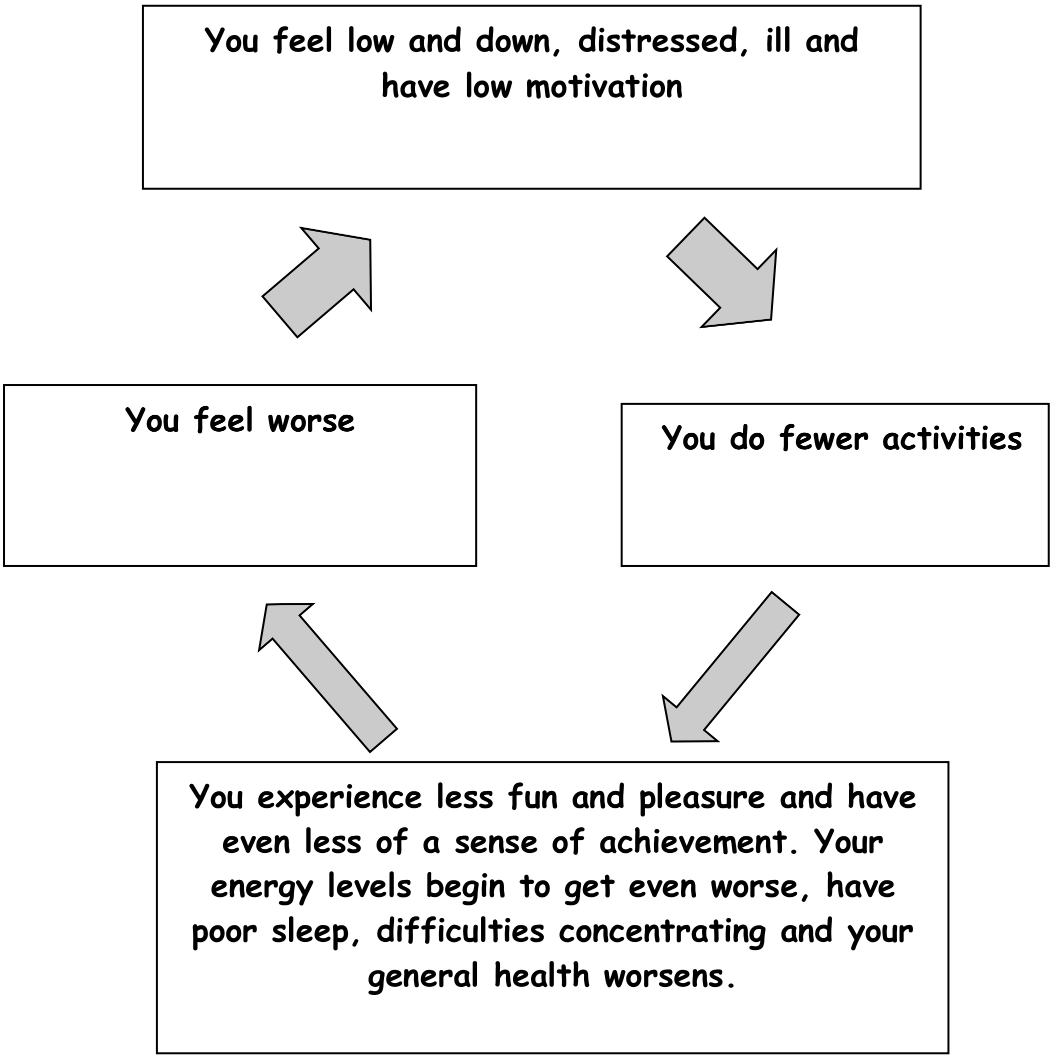

Cycle of low mood and reducing activity

The role of the PAc PWP is to support the participant to increase Physical Activity as part of the BA model. Physical Activity could be described as something that is part of a daily **routine** for example as part of getting somewhere, **pleasurable** (enjoyable in itself, or as part of another task) and **necessary** for improving or maintaining mental and physical health and well-being.

With increases in Physical Activity we anticipate that this will help to maintain benefits arising from BA that have helped to reverse the 'vicious cycle of depression'. As such

tiredness will reduce, thoughts will become more positive, and the participant will start to feel more motivated to do other activities.

The BAcPAc Intervention

What are we asking PWPs to do?

PWPs enrolled in the study are already using a number of taught skills and techniques as part of the successful delivery of BA. These include familiarity with the materials, ensuring the Participant understands the rationale, collaborative patient centred discussion, assisting with appropriate goal setting, and problem solving. These are central to delivery of BAcPAc.

The BAcPAc intervention requires that some differences to normal BA are put in place to help participants to increase their Physical Activity (see the diagram below) . We will be asking BAcPAc PWPs to deliver the intervention by adding an emphasis on Physical Activity by:

1. Providing a clear rationale for how BA works with specific reference to the role of Physical activity
2. Helping the participant to identify a list of possible activities (routine pleasurable and necessary) with use of Worksheet A, and selectively reinforce activities that include physical activity
3. When using Worksheet C to review progress, selectively review progress with activities that involve Physical Activity.
    a. Celebrate/ reflect on success
    b. Normalise setbacks
    c. Check understanding of the BA rationale
    d. Problem solve – may relate to issues of connectedness (social influences)
    e. Where appropriate help to extend physical activity in term of duration, intensity, time and type
4. When the patents shows signs of mood lifting, revisit the rationale with an emphasis on the PA element, exploring other (additional to mood) benefits of PA
5. With the patients agreement, build in self-monitoring of PA using tools the BAcPAc tools (diary keeping with comments, using pedometers and recording steps)

And routinely throughout:

- Review progress with increases in Physical Activity
- Problem solve within the context of BA and PA
- Explore opportunities for social support to increase or maintain physical activity
- Normalise and manage relapse in Physical Activity
- Help to maximise likely enjoyment or utility

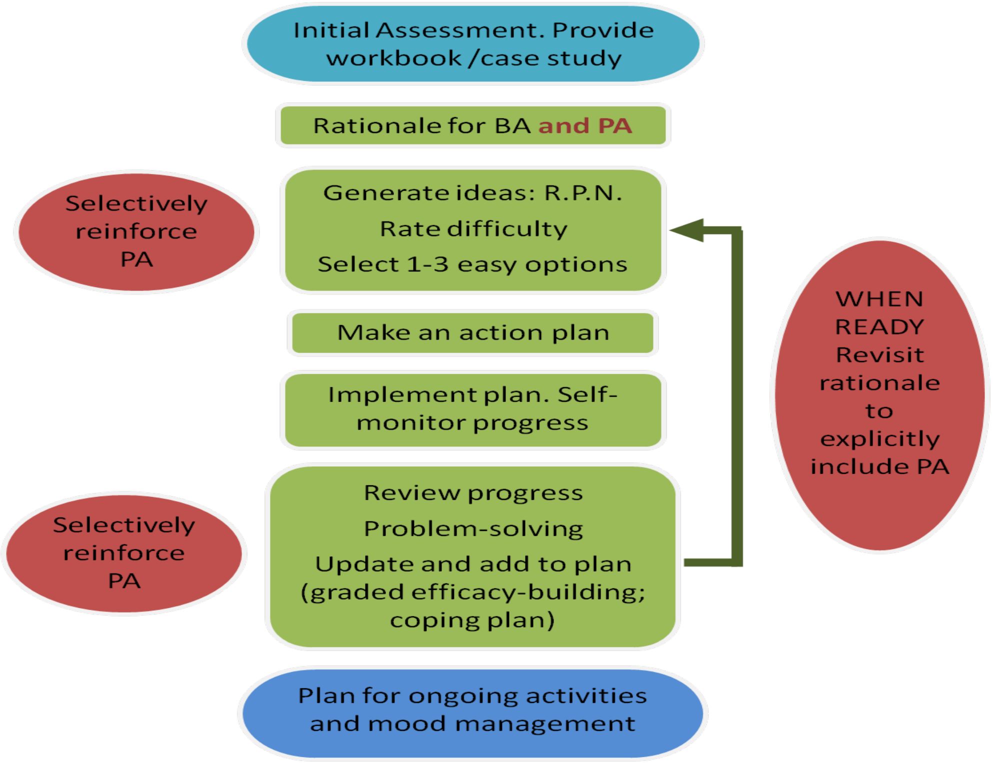

Integrating Physical Activity into normal Behavioural Activation process

(RPN- Routine, Pleasurable, necessary, BA –Behavioural Activation PA –Physical Activity)

Identifying when participants may be ready to increase Physical Activity.

Participants may start to talk about or to plan to do activities that include Physical Activity from the start, or they may need to do some other activities for a couple of weeks for their mood to lift before they start moving around more and being ready to be more physically active. Any talk from the participant about increasing Physical Activity when planning activities for the week should be met with recognition of the inclusion of Physical Activity as part of BA treatment, and a good option. However, be aware of participants expecting to do too much too soon, as with BA.

We expect that when a participant's mood begins to lift they may naturally start to seek to do more activities. This may also be a time when participants are ready to increase more activities that include Physical Activity. PWPs can use the PHQ-9 scores as evidence of a shift in mood, and the worksheets A and B to recognise activities that may include Physical Activity, and to encourage these.

Identifying opportunities to support Physical Activity planning and behaviour

Few people are so completely sedentary that they are not doing any form of Physical Activity. Any Physical Activity that a participant is doing, however small, should be

recognised as a demonstration that the individual is not completely sedentary. This serves to normalise Physical Activity as something that is done by everybody every day. Part of the

---

PWPs will be encouraged to look for any talk from the participant about increasing or adding activities that include some form of Physical Activity, especially when planning activities for the following week. For example a participant may talk about wanting to pay a bill (necessary activity) by walking to the post office. An affirmation of the 'double whammy' benefit of walking to the post office as this is a Physical Activity as well as a necessary activity would be an opportunity to reiterate the BAcPAc rationale.

PWPs role in BAcPAc is to support the participant to recognise the Physical Activity they are doing and to build on this where possible, as well as help the participant to add new activities that include Physical Activity. We would not expect to see a PWP raising the issue of adding Physical Activity with the patient until the patient has started to show signs of engaging with BA and increased mood.

Looking more closely at setting goals for physical activity

Frequency, intensity, timing and type: The F.I.T.T. principle

After Participants have started engaging with BA and start to feel better and would like to increase their physical activity, it is useful to use the F.I. T.T. principle. This is a way of breaking the important parts of the activity down into elements, all of which can be increased separately or together. Each component will have an impact on the potential benefits of the physical activity they are doing.

- **F**requency – How often the activity occurs each week. (ideally most days)
- **I**ntensity – How hard the person is working: How deep and fast the breathing is, how fast their heart rate is. If their breathing increases but they can still hold a conversation that is a good level to aim for which will also be safe
- **T**iming - When the activity is taking place, and the duration of the activity.
- **T**ype- The mode of the activity. For example walking, cycling, digging the garden, mopping the floor.

A note on Type: Choosing some activities that are enjoyable and/or sustainable would be ideal. These can include routine activities such as walking to the shop to get milk, or necessary activities like walking part way to the post office to pay a bill. Thinking about the 3 C's (Confidence, Control and Companionship) is a good way of helping participants to choose the types of pleasurable activity planned in their diary.

Sustainability and relapse

Ideally, participants will be building on existing Physical Activity, and choosing Physical Activities using appropriate goals setting, which will mean the activities should be sustainable, but more than likely there will be times when relapse occurs. The important thing to do will be to normalise relapse, and to treat choosing activities as an experiment, just as you do in normal delivery of BA.

We have included a comments box in the BA diaries to help participants to report any information about difficulties or positive effects of doing the activities, and to help with discussions at PWP sessions. Also working through Worksheet C will help them work through foreseen difficulties of performing the activity.

Using the BAcPAc self- help booklet and case study

You will be supporting the BAcPAc Get Active, Feel Good self-help booklet and using the case studies in the same way that you use the BA manual. There are changes to some of the worksheets and diaries.

The Starting Point Diary and the Next Steps Diary have space for comments at the end of each day. This is to assist with discussions reviewing the previous weeks planned activities and goal setting for the following week. It also provides the option of adding detail to assist with making specific goals – Who with, What activity, Where it will take place.
