# Supplementary material for: A written self-help intervention for depressed adults comparing behavioural activation combined with physical activity promotion with a self-help intervention based upon behavioural activation alone: study protocol for a parallel group pilot randomised controlled trial (BAcPAc)
Source: Trials. 2014 May 29;15:196. doi: 10.1186/1745-6215-15-196 (PMC4061537; doi:10.1186/1745-6215-15-196)
Supplement: Additional file 4 — PWP training manual BA. [file 1745-6215-15-196-S4.pdf]

A study for people with depression or low mood

BAcPAc manual for BA Psychological Wellbeing Practitioners

BAcPAc manual for BA Psychological Wellbeing Practitioner
Version 2
February 2013
Claire Pentecost, Adrian Taylor, Paul Farrand and Colin Greaves
University of Exeter

Contents

The BAcPAc Project: Background 4
Introduction to BAcPAc 5
Study Aim 5
Study Objectives 5
Using the BAcPAc self- help booklet and case study 5
General clinical procedures 7
Frequency and duration of appointments 7
Collecting routine outcome measures 7
Recording sessions 7
Appointments with BAcPAc supervisor 8
Organisation and management 8
Risk protocol 8
Participants randomisation and research being blinded to group allocation 9
Confidentiality and data protection 9

The BAcPAc Project: Background

- By 2020 depression is expected to represent the second highest burden of disease among all general health problems.
- Three quarters of those successfully treated for depression will go on to have at least one more depressive episode.
- Depression and anxiety are estimated to cost the UK economy £17bn in lost output and direct health care costs annually, with a £9bn impact on the Exchequer through benefit payments and lost tax receipts.
- The potential costs of depression may be far higher with the financial burden associated with sustained inactivity that is a key symptom associated with depression, alongside an increased risk of health related problems such as obesity, diabetes and stroke.
- Despite links between mental and physical health, interventions tend to target mental and physical health separately, with little guidance on how best to promote PA within mental health services.

IAPT services

- Psychological Well-being Practitioners (PWPs) are a new workforce created as part of the IAPT (Improving Access to Psychological Therapies) programme implemented across England.
- PWPs undertake a range of evidence based low intensity psychological interventions including Behavioural Activation (BA), which seeks to reduce depressed mood by increasing daily functioning.

Behavioural Activation

- BA is established as an evidence-based treatment for depression.
- Systematic reviews have also demonstrated equal effectiveness of BA for the treatment of depression to other psychological interventions, such as cognitive behavioural therapy (CBT).
- BA seeks to reduce depressed mood by increasing daily functioning.
- Behavioural activation is a technique where participants focus on re-establishing daily routines,increase pleasurable activities and do the things that are necessary for daily living.

Depression and Physical Activity

Studies on PA and depression have mostly involved intensive non-generalisable interventions for the UK. There is no evidence that counselling depressed patients to do more physical activity helps to reduce depression more than usual care and as a PWP who is delivering the control intervention of BA as usual we do not want you to modify what you normally do. Further pragmatic research is needed on how best to integrate Physical Activity into existing low intensity interventions.

Introduction to BAcPAc

Study Aim

To enhance a routine, evidence-based behavioural activation (BA) treatment for people with depression by adding a focus on Physical Activity (PA) behaviour change techniques in a novel intervention, and conduct a pilot RCT to inform the design and delivery of a larger scale randomised controlled trial (RCT).

Study Objectives

Phase II Pilot RCT: (a) Undertake a pilot RCT of BA alone vs. BAcPAc to confirm feasibility and clarify uncertainties in the design and delivery of a larger scale RCT; (b) Assess the acceptability of the BAcPAc intervention amongst participants with depression.

Using the BAcPAc self- help booklet and case study

You will be supporting the BAcPAc Get Active, Feel Good self-help booklet and using the case studies in the same way that you use the BA manual. There are changes to some of the worksheets and diaries.

The Starting Point Diary and the Next Steps Diary have space for comments at the end of each day. This is to assist with discussions reviewing the previous weeks planned activities and goal setting for the following week. It also provides the option of adding detail to assist with making specific goals – Who with, What activity, Where it will take place.
