# Supplementary material for: A written self-help intervention for depressed adults comparing behavioural activation combined with physical activity promotion with a self-help intervention based upon behavioural activation alone: study protocol for a parallel group pilot randomised controlled trial (BAcPAc)
Source: Trials. 2014 May 29;15:196. doi: 10.1186/1745-6215-15-196 (PMC4061537; doi:10.1186/1745-6215-15-196)
Supplement: Additional file 5 — Participant topic guide. [file 1745-6215-15-196-S5.pdf]

BAcPAc Participant Topic guide

General Aims:

- To find out how and why participants became involved
- How acceptable was the data collection
- How acceptable was the intervention
- Beliefs about the usefulness of the intervention – did they increase physical activity and did that help
- What did they like/not like/could be improved about the workbooks and case studies

Intervention Participants		
**Topic**	**Questions**	**Possible prompts**
Opening questions	What did you think about the study when you first heard about it?	Were there any problems with the study information?
	When you were in conversation with Claire (UoE) talking about things such as what the study was about, that you would be put into one of 2 groups, and the information that needed to be collected for the study, was the information about the study clear?	How clear were the explanations of what would be involved?<br>What did you think about being randomised (put into a normal BA group or a modified BA group)
Acceptability of data collection appointments	How was the screening and data collection appointments with Claire?	Is there anything that you would have liked to change about these appointments?
Reasons for taking up/not talking up the intervention	How was it decided what treatment you should receive (ie BA/CBT/Group therapy etc)	Before starting, what treatment did you expect you would receive?<br>Did you have any preferences?<br>How did you/the PWP decide what treatment you should receive?<br>Did the PWP talk to you about the possibility of receiving BA?
Content of the materials and appointments	What were your views about the treatment appointments?	Timing/duration of appointments? Travelling? Style of delivery by PWP?
	Was there anything that did not work so well for you?	
	What did you think about the booklets?	Eg size and length, layout. Description of the treatment, the pace, ranking activities, the diaries, monitoring steps graph (have copy of the book and go through it and at key sections – did this ring true, was this useful?
	Was there anything that you did not like so much/did not find useful?	
	What could be improved about the booklets if anything?	
Style of delivery by the PWP/therapist (use their term)	What did you like about the way the PWP went through the treatment with you?	Listening, demonstrating empathy or understanding, pace, collaborative goal setting, explaining, referring to booklets
	What did you not like so much/did not find useful?	
Increasing activities including physical activity	Did you add more physical activities into your schedule?<br>Did you increase the physical activities that you were doing?	What? How? Why? Why not?
	If Yes What helped you to add more physical activity to what you were already doing?	??Information in materials (understanding benefits, how to), PWP?, help or support from others?, filling in diaries and graphs?, pedometer? something else?
	What else could have been useful?<br>Do you think you will continue with your increased PA?<br>Have you felt any benefits?<br>What may be a reason to start doing more physical activity/start again?	

Relapse	Did you notice any changes in your mood?	IF YES<br>What particular activities helped your mood?<br>What is it about that activity that helped?<br>What would you do if you felt your depression was returning?
Summary	SUMMARISE MAIN POINTS<br>Does that sound about right? Did I miss anything important? Is there anything you want to add?	
