# Supplementary material for: A written self-help intervention for depressed adults comparing behavioural activation combined with physical activity promotion with a self-help intervention based upon behavioural activation alone: study protocol for a parallel group pilot randomised controlled trial (BAcPAc)
Source: Trials. 2014 May 29;15:196. doi: 10.1186/1745-6215-15-196 (PMC4061537; doi:10.1186/1745-6215-15-196)
Supplement: Additional file 6 — BAcPAc intervention fidelity measure. [file 1745-6215-15-196-S6.pdf]

BAcPAc FIDELITY MEASURE

The rating scale

The six point scale (i.e. a 0-6 Likert scale) extends from (0) where the Psychological Wellbeing Practitioner (PWP) did not deliver the intervention element appropriately - either they did not do it well or did not do it sufficiently (low fidelity) to (6) where there is the element is delivered appropriately (high fidelity). Thus the scale assesses a composite of both adherence to the intended intervention techniques and the skill of the PWP in delivering the techniques. To aid with the rating of items, an outline of the key features of each item is provided at the top of each section. A generic description of the rating criteria is given in Figure 1.

Adjusting for the presence of patient difficulties

Adjustments may be needed when patient difficulties are evident (e.g. excessive avoidance or resistance). In such circumstances, the rater needs to assess the PWP's therapeutic skills in the application of the methods. Even though the PWP may not facilitate change, credit should be given for attempting to use the intended techniques and demonstrating appropriate /skilful interaction.

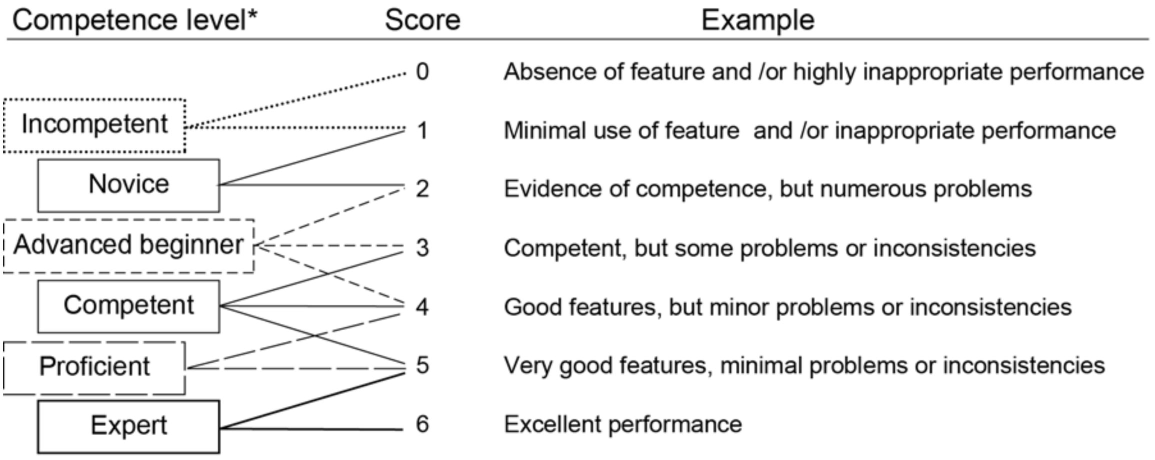

* The scale incorporates the Dreyfus system (Dreyfus, 1989) for denoting competence. Please note that the 'top marks (i.e. near the 'expert' end of the continuum) are reserved for those PWPs demonstrating highly effective skills, particularly in the face of difficulties (i.e. patients with high resistance to change; high levels of emotional expression; and complex situational barriers). Please note that there are 5 competence levels but six potential scores.

When rating the item, you should first identify whether some of the 'Key Features' are present. If the PWP includes most of the key features and uses them appropriately (i.e. misses few relevant opportunities to use them and delivers them well), the PWP should be rated very highly. It is important to remember that the scoring profile for this scale should approximate to a normal distribution, with relatively few scoring at the extremes.

Dreyfus, H. L. (1989). The Dreyfus model of skill acquisition. In J. Burke (ed.) Competency based education and training. London: Falmer Press.

ITEM 1: ACTIVE PATIENT INVOLVEMENT

Key features: The PWP should encourage the participant to be actively involved in the consultation. The idea is to maximise the participant's autonomy as the main agent of change, developing intrinsic rather than extrinsic motivation, and encouraging her /him to be the person coming up with ideas for improving the situation. However, the participant should not be allowed to ramble in an unstructured way and the consultation should be guided. A collaborative /shared decision-making style is appropriate and the PWP may share his /her own expertise and ideas, using techniques such as elicit-provide-elicit (below). Overall, the participant should be increasingly empowered to take control of her /his physical activity behaviour. Interactions should be encouraging, respectful and non-judgemental (the opposite of a didactic, telling or persuading style of interaction). The participant should ideally talk for at least half of the time. The interaction should also be *individually tailored* to the patient's specific information needs, beliefs, motivations and barriers. The PWP should engender a clear sense of warmth, genuineness and empathy (within professional boundaries).

Intervention techniques: OARS (Open questions, Affirmation, Reflective listening, Summaries). Reflective listening may include simple reflections of content but may also be more sophisticated (e.g. amplified reflection; reflection with a twist) and used to direct the conversation or highlight key strengths or barriers. Summaries to reinforce patient choices and acknowledge patient effort are particularly desirable.

Individual tailoring of techniques and responses to the individual patient's existing knowledge, skills, current activity levels, needs and preferences are also desirable.

The Ask-Tell-Discuss (elicit-provide-elicit) technique should be used to exchange information (e.g. to address misconceptions, or offer helpful new information such as the rationale for BA (or BAcPAc), or the benefits of physical activity). The above empathy-building techniques and Individual tailoring should be used throughout the consultations - from the initial consultation through action-planning through to review /maintenance sessions.

Using whole and half numbers, indicate the level to which you think the PWP has delivered this intervention process

0 Absence of active patient involvement techniques. A highly didactic /practitioner-led or ‘lecturing’ style of interaction, which may increase or sustain client’s resistance

1 Minimal patient involvement or use of active patient involvement techniques. The practitioner dominates the discussion

2 Appropriate use of patient involvement techniques, but not frequent enough. The practitioner sometimes dominates the discussion

3 Appropriate and frequent use of patient involvement techniques. Teamwork evident, but some difficulties in content or method of delivery

4 Appropriate and frequent use of patient involvement techniques. Minor problems evident (e.g. some reflection opportunities missed)

5 Highly appropriate and regular use of patient involvement techniques, facilitating shared understanding and decision making. Minimal problems

6 Excellent / expert use of patient involvement techniques throughout all consultations. A clear sense of collaborative alliance is developed.

ITEM 2a: PROVIDE A CLEAR RATIONALE FOR HOW BEHAVIOURAL ACTIVATION WORKS

Key features: The PWP should work with the participant to explain how behavioural activation works by showing the participant the diagram and checking the participants understanding of it. Participants' 'buy in' will be crucial for the success of the intervention as belief in the benefit of the method will aid motivation to complete homework and activities.

Intervention techniques: PWPs will provide the BA (or BAcPAc) booklet as "homework" (specifically to read the case study). In subsequent sessions, they should use patient-centred information-exchange techniques (i.e. Ask-Tell-Discuss or 'elicit-provide-elicit') to explore existing understanding of how Behavioural Activation works and to identify and address any misconceptions. The case study (which includes an implicit explanation of the BA rationale) may be referred to help illustrate how BA works. The rationale should be explicitly discussed and referred back to /reinforced at subsequent sessions.

Using whole and half numbers, indicate the level to which you think the PWP has delivered this intervention process

0 Absence (or very poor delivery of) explanation and discussion of the rationale of how behavioural activation works. Understanding of the rationale is assumed or not mentioned or discussed

1 Minimal (or poor delivery of) explanation and discussion and checking understanding of the rationale of how behavioural activation works.

2 Some discussion of the rationale, but the explanation is not of sufficient depth or detail or understanding is not checked.

3 Appropriate explanation and discussion of the rationale for how behavioural activation works. However, some difficulties evident (e.g. moving on before understanding is fully established)

4 Appropriate explanation and discussion of the rationale for how behavioural activation works. Appropriate and frequent use of the rationale across several sessions, linking changes in mood with changes in behaviour. Minor problems evident (e.g. some inconsistencies)

5 Highly appropriate and sufficient discussion of the rationale across several sessions, facilitating a clear understanding of the BA process and linking changes in mood with changes in behaviour. Minimal problems

6 Excellent / expert use of the rationale, facilitating a clear understanding of reasons for change. No real problems

ITEM 2b: GO BEYOND 2a TO PROVIDE A CLEAR EXPLANATION OF *THE SPECIFIC ROLE OF PHYSICAL ACTIVITY* IN BEHAVIOURAL ACTIVATION

Key features: As part of working with the participant to explain how behavioural activation works (see item 2a), the PWP should make specific reference to the role of physical activity, emphasising its contribution in relation to the diagram in the intervention booklet and checking the participants understanding of it. The physical activity element should be explicitly discussed and referred back to at subsequent sessions.

Intervention techniques:

PWPs should use patient-centred information-exchange techniques (i.e. Ask-Tell-Discuss or ‘elicit-provide-elicit’) to explore understanding of the particular role of physical activity in Behavioural Activation /in improving mood, and to identify and address any misconceptions. The case study (which includes an implicit explanation of the BAcPAc rationale) may be referred to help illustrate the role of physical activity. The role of physical activity should be explicitly discussed and referred back to /reinforced at subsequent sessions.

Using whole and half numbers, indicate the level to which you think the PWP has delivered this intervention process

0 Absence (or very poor delivery) of the explanation discussion and checking understanding, of the role of physical activities in improving mood. Understanding of the role of physical activity) is assumed or not mentioned or discussed

1 Minimal (or poor delivery of) explanation discussion and checking understanding, of the role of physical activities in improving mood. Inadequate or non-specific discussion of the role of physical activities in improving mood

2 Some explanation and discussion and checking understanding of the role of physical activities in improving mood, but the explanation is not of sufficient depth or detail or understanding is not checked.

3 Appropriate explanation and discussion of the role of physical activities in improving mood. However, some difficulties evident (e.g. moving on before understanding is fully established)

4 Appropriate explanation and discussion of the role of physical activities in improving mood. Appropriate and frequent referral to the role of PA across several sessions, linking changes in mood with changes in behaviour. Minor problems evident (e.g. some inconsistencies)

5 Highly appropriate and sufficient discussion of the role of physical activity in improving mood across several sessions, facilitating a clear understanding and linking changes in mood with changes in behaviour, especially physical activity behaviour. Minimal problems

6 Excellent / expert explanation and discussion of the role of physical activity in improving mood across several sessions, facilitating a clear understanding of reasons for change. No real problems

ITEM 3a: IDENTIFY POSSIBLE ACTIVITIES, SELECTING THEM TO MAXIMISE BOTH THE EASE OF INITIAL ADOPTION AND THE SUSTAINABILITY OF THE ACTIVITIES

Key features: Using worksheet A in the intervention manual the PWP should work with the participant to identify a range of activities to engage in. The PWP should help the participant to identify activities that are: Routine, Pleasurable or Necessary. They should use Worksheet B to rank chosen activities in terms of difficulty. They should agree a verbal plan of action, seeking to choose the easiest options first.

Intervention techniques: OARS (Open questions, Affirmation, Reflective listening, Summaries) should be used specifically to elicit ideas and explore the types of activities that the participant used to do or would like to do. Patient choices are affirmed and summarised to reinforce the choice of activities. Action ideas should be filtered, using worksheets A and B to meet the RPN criterion and to identify the easiest options.

Mark with an 'X' on the vertical line, using whole and half numbers, the level to which you think the PWP has delivered this intervention process

0 Absence (or very poor delivery) of an attempt to engage the patient in working through Worksheet A, to choose activities that are routine, pleasurable and necessary, and ranking activities in order of difficulty.

1 Minimal (or poor delivery) of an attempt to engage the patient in working through Worksheet A, or a lack of a patient centred counselling techniques to choose activities that are routine, pleasurable and necessary, and ranking activities in order of difficulty.

2 Some attempt to engage the patient in working through Worksheet A, and choosing activities that are routine, pleasurable and necessary according, and then ranking according to difficulty using patient centred counselling techniques. However the discussion may be driven by PWP rather than the patient, or discussion of possible activities is not in sufficient depth).

3 Appropriate engagement and working through Worksheet A using patient centred counselling techniques to choose routine pleasurable and necessary activities, and or ranking activities in order of difficulty. However, some difficulties evident (e.g. sustainability issues (RPN or ranking processes) not fully discussed).

4 Appropriate engagement and working through Worksheet A using patient centred counselling techniques to choose routine pleasurable and necessary activities, and or ranking activities in order of difficulty. Minor problems evident (e.g. the chosen activities have not been summarised).

5 Highly appropriate and sufficient use of Worksheet A and patient centred counselling techniques to choose routine, pleasurable and necessary activities, and ranking activities in order of difficulty. Activities chosen are agreed between the PWP and participant and have been filtered according to sustainability. Minimal problems.

6 Excellent / expert use of Worksheet A and patient centred counselling techniques to choose activities that are routine, pleasurable and necessary, ranking activities according to difficulty and choosing the easiest first. No real problems.

ITEM 3b: GO BEYOND 3a TO SPECIFICALLY REINFORCING PHYSICAL ACTIVITY

Key features: When using worksheet A in the BA intervention manual, the PWP should work with the participant to specifically identify some activities that include physical activity. The PWP should selectively reinforce any physical activities that are mentioned and then help the participant to identify activities that include physical activity that can be categorised as: Routine, Pleasurable and Necessary. If appropriate, they should encourage incorporation of physical activity related items in the verbal plan of action. .

Intervention techniques: OARS (Open questions, Affirmation, Reflective listening, Summaries) should be used specifically to elicit ideas and explore the types of physical activities that the participant used to do or would like to do. Patient choices that include physical activity should be selectively affirmed and reinforced with reference to the rationale where appropriate.

Using whole and half numbers, indicate the level to which you think the PWP has delivered this intervention process

0 Absence (or very poor delivery) of an attempt to specifically identify and reinforce physical activity items when working through Worksheet A.

1 Minimal (or poor delivery) of an attempt to specifically identify and reinforce physical activity items when working through Worksheet A, or a lack of a patient centred counselling techniques when doing this.

2 Some attempt to specifically identify and reinforce physical activity items when working through Worksheet A, using patient centred counselling techniques. However the discussion may be driven by PWP rather than the patient, or discussion of the physical activity element is not in sufficient depth.

3 Appropriate engagement to specifically identify and reinforce physical activity items when working through Worksheet A, using patient centred counselling techniques. However, some difficulties evident (e.g. PWP misses several opportunities to reinforce or highlight the physical activity component).

4 Appropriate engagement to specifically identify and reinforce physical activity items when working through Worksheet A using patient centred counselling techniques. Minor problems evident (e.g. the PA component of the chosen activities has not been summarised).

5 Highly appropriate and sufficient use of techniques to specifically identify and reinforce physical activity items when using Worksheet A, including patient centred counselling techniques. Minimal problems.

6 Excellent / expert use of techniques to specifically identify and reinforce physical activity items when using Worksheet A, including excellent use of patient centred counselling techniques. No real problems.

ITEM 4: MAKE A SPECIFIC ACTION PLAN, BASED ON THE ACTIVITIES SELECTED BY THE PATIENT

Key features: The PWP should work with the participant to agree a written plan of action for the following week, being as specific as possible by using the Next Steps diary.

Intervention techniques: Making a written action plan, using the Next Steps diary in the manual. Action plans should be specific including elements of 'What, Where and Who with". The PWP should ensure that goal-setting is realistic (no more than 3-4 changes). The PWP may also employ some problem-solving techniques at this stage to pre-empt and address potential problems (this is good practice, but was not incorporated in the training, so should not be expected)

Using whole and half numbers, indicate the level to which you think the PWP has delivered this intervention process

0 Absence (or very poor delivery) of action planning / scheduling activities for the following week.

1 Minimal use (or poor delivery) of action planning / scheduling activities for the following week. Activities planned are not sustainable, or representative of the routine, pleasurable and necessary activities previously identified.

2 Some use of action-planning techniques using the next steps diary but lacking detail /patient involvement in the activity may be limited.

3 Appropriate use of action planning techniques and discussion of the use of the next steps diary. However, some difficulties evident (e.g. not explaining the importance of using the diary as a basis for self-monitoring, providing rather than eliciting ideas)

4 Appropriate use of action planning techniques and discussion of the use of the next steps diary. Minor problems evident (e.g. the plan is a bit less specific than it could be)

5 Highly appropriate and sufficient use of action-planning techniques, discussion of and active completion of the next steps diary. The participant has a clear understanding of the plan for the week ahead. Minimal problems

6 Excellent / expert use of action-planning techniques, and discussion of and active completion of the next steps diary. The participant has a clear understanding of the rationale behind planning for the week ahead, and has a clear and realistic action plan for the week ahead. No real problems

ITEM 5: INITIATING THE TARGETED NEW ACTIVITIES/ESTABLISHING SELF-MONITORING

Key features: The PWP should discuss initiating the plan of action for the following week by setting this as ‘homework’ and referring to the Next Steps Diary. He /she should discuss the use of the diary to self-monitor and to keep track of progress and as a way of recording any problems immediately following completing an activity/task.

Intervention techniques: to the PWP should encourage the participant to monitor /keep track of their activities using the Next Steps Diary.

Using whole and half numbers, indicate the level to which you think the PWP has delivered this intervention process

0 Absence (or very poor delivery) of encouragement to try out the planned activities over the next week and to use the Next Steps Diary to monitor completion of the planned activities

1 Minimal (or poor delivery) encouragement to try out the planned activities over the next week and to use the Next Steps Diary to monitor completion of the planned activities

2 Some encouragement to try out the planned activities over the next week and to use the Next Steps Diary to monitor completion of the planned activities but lacking sufficient depth or detail (this can be quite a brief discussion however)

3 Appropriate encouragement to try out the planned activities over the next week and to use the Next Steps Diary to monitor completion of the planned activities. However, some difficulties evident (e.g. providing instruction without negotiation)

4 Appropriate encouragement to try out the planned activities over the next week and to use the Next Steps Diary to monitor completion of the planned activities. Minor problems evident

5 Highly appropriate and sufficient encouragement to try out the planned activities over the next week and to use the Next Steps Diary to monitor completion of the planned activities, facilitating a clear understanding of the usefulness of self-monitoring. Minimal problems

6 Excellent / expert encouragement to try out the planned activities over the next week and to use the Next Steps Diary to monitor completion of the planned activities facilitating a clear understanding of the usefulness of self-monitoring. No real problems

ITEM 6a: REVIEWING PROGRESS, IDENTIFYING AND ADDRESSING ANY PROBLEMS ENCOUNTERED AND SEEKING TO PREVENT RELAPSE

Key features: The PWP should work with the participant to review progress in general with depressed mood (by completing the PHQ9) and with achieving the behavioural targets set out in the action plan. The PWP should celebrate and reinforce and reflect on any successes. The participant and PWP should discuss any setbacks and the action plan should be revised, with an attempt to extend activities. If there is no progress after 2-3 weeks, different therapies may be discussed.

Intervention techniques: The PHQ9 results are reflected upon in relation to progress in general. The PWP should reinforce any self-monitoring, successes in behaviour change. Reframing should be used to normalise setbacks and see them as an opportunity to learn from experience (trial and error) rather than as failures. Problem-solving should use OARS (Open questions, Affirmation, Reflective listening, Summaries) to identify barriers and explore ways to overcome them (problem-solving). Problem solving may specifically relate to issues of connectedness (social influences, involvement of others in activities) and sustainability. The BA rationale may be referred back to and reinforced (but code this under item 2). Goals /action plans should be reviewed and revised if necessary.

Using whole and half numbers, indicate the level to which you think the PWP has delivered this intervention process

0 Absence (or very poor delivery) of any progress review. No reinforcement of success and discussion of setbacks or barriers in relation to the previous weeks planned activities /problem-solving, or reviewing the action plan for the following week.

1 Minimal (or poor delivery) of progress review. Minimal reinforcement of success and discussion of setbacks or barriers in relation to the previous weeks planned activities /problem-solving, or reviewing the action plan for the following week.

2 Some progress review. Some reinforcement of success and discussion of setbacks or barriers in relation to the previous weeks planned activities /problem-solving, and reviewing the action plan for the following week, but lacking sufficient depth or detail.

3 Appropriate progress review. Appropriate reinforcement of success and discussion of setbacks or barriers in relation to the previous weeks planned activities /problem-solving, and reviewing the action plan for the following week. However, some difficulties evident (e.g. not reframing setback, not attempting to identify problems, or possible solutions).

4 Appropriate progress review. Appropriate reinforcement of success and discussion of setbacks or barriers in relation to the previous weeks planned activities /problem-solving, and reviewing the action plan for the following week. Minor problems evident.

5 Highly appropriate and sufficient progress review. Appropriate reinforcement of success and discussion of setbacks or barriers in relation to the previous weeks planned activities /problem-solving, or reviewing the action plan for the following week. Minimal problems

6 Excellent / expert progress review. Excellent reinforcement of success and discussion of setbacks or barriers in relation to the previous weeks planned activities /problem-solving, and reviewing the action plan for the following week. No real problems

ITEM 6b: REVIEWING PROGRESS, IDENTIFYING AND ADDRESSING ANY PROBLEMS & RELAPSE PREVENTION, SPECIFICALLY RELATING TO PHYSICAL ACTIVITY

Key features: In addition to the above progress review, the PWP should work with the participant to specifically review progress with activities that involve physical activity. Any successful activity that includes physical activity should be reinforced /affirmed and reflected on in particular. The participant and PWP should discuss any setbacks and the action plan should be revised, with an attempt to extend any PA. If there is no progress after 2-3 weeks, different therapies may be discussed.

Intervention techniques: The PWP should reinforce any successes in physical activity-related behaviour change (particularly noting the PA element). Reframing should be used to normalise setbacks. Problem-solving should use OARS (Open questions, Affirmation, Reflective listening, Summaries) to identify barriers and explore ways to overcome them (problem-solving). The role of physical in the BA rationale may be reflected on /reinforced (but code this under item 2b). Goals /action plans specific to increasing one or more activity that involves physical activity should be reviewed and revised if necessary, considering duration, intensity frequency and type.

Using whole and half numbers, indicate the level to which you think the PWP has delivered this intervention process. It is recognised that there may only be a few, if any opportunities to deliver this aspect of the intervention. Hence, we expect scores to be relatively low for this item.

0 Absence (or very poor delivery) of any progress review. No reinforcement of success or discussion of setbacks or barriers in relation to the previous weeks planned activities that include PA, or reviewing the action plan for the following week to incorporate PA.

1 Minimal (or poor delivery) progress review. Minimal reinforcement of success and discussion of setbacks or barriers in relation to the previous weeks planned activities that include PA, or reviewing the action plan for the following week to incorporate PA.

2 Some progress review. Some reinforcement of success and discussion of setbacks or barriers in relation to the previous weeks planned activities that include PA, or reviewing the action plan for the following week to incorporate PA but lacking sufficient depth or detail.

3 Appropriate progress review. Appropriate reinforcement of success and discussion of setbacks or barriers in relation to the previous weeks planned activities that include PA, and reviewing the action plan for the following week to incorporate PA. However, some difficulties evident (e.g. not specifically reinforcing activities that included physical activity, providing rather than eliciting possible solutions to problems)

4 Appropriate progress review. Appropriate reinforcement of success and discussion of setbacks or barriers in relation to the previous weeks planned activities that include PA, or reviewing the action plan for the following week to incorporate PA. Minor problems evident

5 Highly appropriate and sufficient progress review. Highly appropriate reinforcement of success and discussion of setbacks or barriers in relation to the previous weeks planned activities that include PA, and reviewing the action plan for the following week to incorporate PA. Minimal problems

6 Excellent / expert progress review. Excellent reinforcement of success and discussion of setbacks or barriers in relation to the previous weeks planned activities that include PA, and reviewing the action plan for the following week to incorporate PA. The participant has a clear understanding of how to move forward. No real problems.

ITEM 7b: WHEN THE PATIENT'S DEPRESSION BEGINS TO LIFT, RE-VISIT PA AND SPECIFICALLY EXPLORE AND SEEK TO ENHANCE THE PATIENTS' WILLINGNESS TO INCREASE THE AMOUNT OF PHYSICAL ACTIVITY GOING FORWARD.

Key features: When the PWP judges that depression has started to lift the PA rationale should be revisited. Other benefits of physical activity (in addition to improved mood) should be explored.

Intervention techniques: Where mood has improved (based on PHQ9 assessment), the PWP should revisit the rationale, with a specific emphasis on the PA element. Using open questions, affirmation and reflective listening, the PWP should provide information about the link between PA and improved mood and explores other benefits of increased PA. The PWP should seek agreement from the patient to specifically target PA as part of their action plan from this point forward. Any barriers to PA should be problem solved /broken down (with use of Worksheet C).

Using whole and half numbers, indicate the level to which you think the PWP has delivered this intervention process

0 Absence (or very poor delivery) of revisiting the role of PA (and its wider benefits) when mood has lifted and seeking agreement to target PA specifically going forward.

1 Minimal (or poorly delivered) revisiting role of PA (and its wider benefits) when mood has lifted and seeking agreement to target PA specifically going forward.

2 Some revisiting of the role of PA (and its wider benefits) when mood has lifted and seeking agreement to target PA specifically going forward. but not in sufficient depth or detail.

3 Appropriate revisiting of the role of PA (and its wider benefits) when mood has lifted and seeking agreement to target PA specifically going forward. However, some difficulties evident (e.g. missed opportunities, patient being pushed, rather than guided).

4 Appropriate revisiting of role of PA (and its wider benefits) when mood has lifted and seeking agreement to target PA specifically going forward. Minor problems evident.

5 Highly appropriate and sufficient revisiting of the role of PA (and its wider benefits) when mood has lifted and seeking agreement to target PA specifically going forward. Minimal problems.

6 Excellent / expert revisiting of the role of PA (and its wider benefits) when mood has lifted and seeking agreement to target PA specifically going forward. No real problems.

ITEM 8b: SYSTEMATICALLY BUILD PHYSICAL ACTIVITY

Key features: With the patients agreement the revised action plan will seek to either a) increase any existing PA behaviour b) Add PA related activities into the patients plan. Self-monitoring with the use of pedometers may be introduced, and reducing sedentary time (as opposed to increasing PA) May be introduced as part of the action plan. Barriers to PA should be explored using Worksheet C and the physical activity specific actions plan and self-monitoring diary (Worksheets D and E) may also be brought into use.

Intervention techniques: Action-planning techniques and Open questions, Affirmation, Reflective listening and Summaries may be used to encourage the participant to increase the amount of sustainable Physical Activity by increasing duration, intensity frequency or type of PA. Progress of planned activities including PA should be reviewed with problem solving and reviewing of action plans to increase PA going forward. The use of Pedometers and/or the BA diary to self-monitor should be explained and explored and incorporated into the action plan if appropriate.

Mark with an 'X' on the vertical line, using whole and half numbers, the level to which you think the PWP has delivered this intervention process

0 Absence (or very poor delivery) of discussions, planning, encouraging self-monitoring, progress review, problem-solving and review of action plans to increase existing PA or add new PA related activities.

1 Minimal (or poorly delivered) discussions, planning, encouraging self-monitoring, progress review, problem-solving and review of action plans to increase existing PA or add new PA related activities.

2 Some discussions, planning, encouraging self-monitoring, progress review, problem-solving and review of action plans to increase existing PA or add new PA related activities, but not in sufficient depth or detail.

3 Several examples of discussions, planning, encouraging self-monitoring, progress review, problem-solving and review of action plans to increase existing PA or add new PA related activities. However some difficulties evident (e.g. missed opportunities to review or problem-solve PA, not explaining the importance of self-monitoring or talking at odds with the patient).

4 Several examples of discussions, planning, encouraging self-monitoring, progress review, problem-solving and review of action plans to increase existing PA or add new PA related activities. Minor problems evident.

5 Highly appropriate and sufficient discussions, planning, encouraging self-monitoring, progress review, problem-solving and review of action plans to increase existing PA or add new PA related activities. Minimal problems.

6 Excellent / expert use of discussions, planning, encouraging self-monitoring, progress review, problem-solving and review of action plans to increase existing PA or add new PA related activities. No real problems.

ITEM 9a: BRINGING THE THERAPY TO A CLOSE

Key features: Progress should be consolidated and reinforced. Plans for long-term sustainability of activities and of strategies learned for managing depression should be discussed.

Intervention techniques: The PWP will review progress since the start of therapy, reinforce what has been learnt. The rationale should be reviewed. Useful strategies that were helpful should be identified. Plans to stay well /prevent relapse should be discussed using the 'Low mood alarm and activity toolkit' and 'cues for action' and plans to revisit the workbook in the future if depression starts to return should be discussed. The PWP will discuss plans to sustain any new activities, identifying any potential problems and coping strategies to overcome these. The possibility of good and bad days should be discussed and normalised.

Mark with an 'X' on the vertical line, using whole and half numbers, the level to which you think the PWP has delivered this intervention process

0 Absence (or very poor delivery) of discussions to bring the therapy to a close. Not considering progress and long term planning using the above strategies.

1 Minimal (or poorly delivered) of discussions to bring the therapy to a close. Minimal consideration of progress and long term planning using the above strategies.

2 Some of discussions to bring the therapy to a close. Some consideration of progress and long term planning using the above strategies, but not in sufficient depth or detail.

3 Appropriate discussions to bring the therapy to a close. Appropriate consideration of progress and long term planning using the above strategies. However some difficulties evident (e.g. missed opportunities to reinforce what has been learnt to manage mood, PWP dominating the conversation /telling rather than guiding).

4 Several examples of appropriate discussions to bring the therapy to a close and examples of consideration of progress and long term planning the above strategies. Minor problems evident.

5 Highly appropriate and sufficient discussion to bring the therapy to a close and to consider progress and long term planning using the above strategies. Minimal problems.

6 Excellent / expert discussions to bring the therapy to a close and to consider progress and long term planning using the above strategies. No real problems.

Item 9b: BRINGING THE THERAPY TO A CLOSE: PHYSICAL ACTIVITY AS A STRATEGY SHOULD BE SPECIFICALLY INCLUDED.

Key features: Alongside the above therapy-closing activities (9a), progress with physical activity goals should be consolidated and reinforced. Plans for long-term sustainability of physical activities and their relevance for managing depression should be discussed.

Intervention techniques: The PWP will review progress since the start of therapy, reinforcing what has been learnt about the role of physical activity in particular. The PA element of the rationale should be reviewed, reinforcing the usefulness of physical activity as a preventative as well as a treatment strategy. Useful strategies that were helpful that included PA should be identified using the 'Low mood alarm and activity toolkit' and plans to plan to use PA again in the future if depression starts to return. Using OARS techniques the PWP will discuss plans to sustain any new physical activities, and pre-empt future problems by making a coping plan for PA in particular (which may be part of the plan for sustaining general activities).

Mark with an 'X' on the vertical line, using whole and half numbers, the level to which you think the PWP has delivered this intervention process

0 Absence (or very poor delivery) of the topic of physical activity in actions to bring the therapy to a close. Not considering progress and long term planning in relation to physical activity using the above strategies.

1 Minimal (or poorly delivered) inclusion of the topic of physical activity in actions to bring the therapy to a close. Minimal consideration of progress and long term planning in relation to physical activity using the above strategies.

2 Some inclusion of the topic of physical activity in actions to bring the therapy to a close. Some consideration of progress and long term planning in relation to physical activity using the above strategies, but not in sufficient depth or detail.

3 Appropriate inclusion of the topic of physical activity in actions to bring the therapy to a close. Appropriate consideration of progress and long term planning in relation to physical activity using the above strategies. However some difficulties evident (e.g. missed opportunities to reinforce the use of PA to manage mood).

4 Several examples of inclusion of the topic of physical activity in actions to bring the therapy to a close and examples of consideration of progress and long term planning in relation to physical activity using the above strategies. Minor problems evident.

5 Highly appropriate and sufficient inclusion of the topic of physical activity in actions to bring the therapy to a close by considering progress and long term planning in relation to physical activity using the above strategies. Minimal problems.

6 Excellent / expert inclusion of the topic of physical activity in actions to bring the therapy to a close by considering progress and long term planning in relation to physical activity using the above strategies. No real problems.

Item 10: DELIVERING THE OUTCOME MEASURES EVERY SESSION

Key features: completing questionnaires on mood (PHQ-9) and other outcomes required by the BA manual

Intervention techniques: n/a.

Did the therapist complete all of the required questionnaires?

Yes ☐ No ☐
